# KCC2 activation during postnatal development alleviates long-term deficits in CDKL5-deficient mice

**DOI:** 10.1038/s12276-026-01670-x

**Published:** 2026-02-19

**Authors:** Muhammad Nauman Arshad, Christopher Bope, Noell Cho, Jacob S. Dengler, Shu Fun Josephine Ng, Joshua L. Smalley, Toshiya Nishi, Zhong Zhong, Stephen J. Moss, Paul A. Davies

**Affiliations:** 1 Department of Neuroscience, Tufts University School of Medicine, Boston, MA USA; 2 Ovid Therapeutics, New York, NY USA; 3 Department of Neuroscience, Physiology and Pharmacology, University College London, London, UK

**Keywords:** Developmental disorders, Autism spectrum disorders, Translational research, Drug development, Autism spectrum disorders

## Abstract

Cyclin-dependent kinase-like 5 (CDKL5) deficiency disorder (CDD) is a severe developmental and epileptic encephalopathy characterized by early onset drug-resistant seizures and later cognitive and social impairments. Existing therapies primarily involve antiseizure medications, which have sedative side effects and lack effective treatments for behavioral impairments. Potassium chloride cotransporter (KCC2) activity is regulated by phosphorylation and is a crucial component of the GABAergic inhibitory system. However, KCC2 dysfunction in CDD remains poorly understood. Here, to investigate potential KCC2 dysfunction, we used a constitutive *Cdkl5* knockout mouse model of CDD. We used liquid chromatography coupled with tandem mass spectrometry and quantitative analysis to examine the unbiased phosphorylation of KCC2. We observed aberrant KCC2 phosphorylation and reduced expression, suggesting reduced KCC2 activity. Examining developmental KCC2 changes revealed significant alterations in key phosphorylation residues and decreased expression between postnatal days 14 and 21. Treatment with the KCC2 activator (OV350) between p10 and p21 saw a significant reduction in infantile spasms compared to vehicle-treated *Cdkl5* knockout mice. Remarkably, when these mice were adults, the mice that received OV350 as pups had reduced seizure susceptibility and their cognitive and behavioral deficits were alleviated. These findings indicate that enhancing KCC2 function during a critical developmental window may be a promising therapeutic strategy for CDD and other developmental and epileptic encephalopathies.

## KCC2 activation alleviates cognitive deficits in CDD mice

Cyclin-dependent kinase-like 5 (CDKL5) deficiency disorder (CDD) is a severe brain disorder causing seizures and developmental issues in children. Here researchers explored a new approach using a molecule called OV350 to enhance the activity of a protein called KCC2, which helps regulate brain cell activity. The researchers used mice lacking CDKL5 to mimic human CDD. They treated these mice with OV350 during a critical development period to see if it could improve symptoms. The study involved both male and female mice, and the treatment aimed to boost KCC2 activity, which is crucial for normal brain function. The results showed that OV350 treatment reduced seizure severity and improved social behavior and memory in adult mice. This suggests that enhancing KCC2 activity during early development can help mitigate some CDD symptoms.

This summary was initially drafted using artificial intelligence, then revised and fact-checked by the author.

## Introduction

Cyclin-dependent kinase-like 5 (Cdkl5) deficiency disorder (CDD) is a severe type of neurodevelopmental and epileptic encephalopathy (DEE) that affects 1 in 40,000–75,000 live births, and is one of the most common genetic forms of infantile epilepsy^1^. CDD is characterized by early onset treatment-resistant seizures beginning in early childhood, accompanied by severe neurodevelopment impairments and subsequent cognitive and social deficits throughout life^2,3^. Treatment-resistant seizures substantially raise the risk of injury or death^4^, highlighting the importance of understanding the underlying mechanisms and developing effective treatments. Current pharmacological therapies for CDD primarily focus on antiseizure medications (ASMs), such as the GABAergic enhancing neuroactive steroid, ganaxolone^5^. However, ASMs (especially when given as polytherapy) are associated with side effects such as somnolence, which limit the quality of life^6,7^. Unfortunately, there is no effective treatment for the cognitive or behavioral impairments associated with this disorder.

Most of the neuropathological changes and behavioral deficits that occur in human patients with CDD can be recapitulated in constitutive *Cdkl5* knockout (KO) mouse models. Like human patients, loss of functional CDKL5 results in increased anxiety, depression and fear-related behavior, along with impairment in the acquisition and retention of spatial memory^8–10^. These *Cdkl5* KO mice also show abnormal adult neurogenesis, reduced dendritic arborization and disruption in the organization of excitatory and inhibitory synapses^11,12^. Recently, a study showed a reduction in the protein levels of phosphorylated K^+^/Cl^−^ cotransporter 2 (KCC2) in the cortex of neonatal *Cdkl5* KO pups, which leads to the development of spontaneous recurrent seizures^13^.

CDKL5 is a serine/threonine protein kinase and is known to be essential for normal brain development. Postnatal developmental expression of CDKL5 has a similar timeline for the developmental activity of the potassium chloride cotransporter (KCC2). During this initial postnatal period, dramatic changes occur as major neuronal circuits are formed that lay down the initial pathways important for memory consolidation and sensory and behavioral processing^14^. KCC2 is the principal Cl^−^-extrusion mechanism used by developing and mature neurons in the central nervous system^15^. Its activity is a prerequisite for the efficacy of fast synaptic inhibition mediated by g-aminobutyric acid type A receptors (GABA_A_R), which are Cl^−^-permeable ligand-gated ion channels. The postnatal development of canonical hyperpolarizing GABA_A_R currents reflects the progressive decrease of intraneuronal Cl^−^ levels, caused by the upregulation of KCC2 expression and subsequent activity^16^. The developmental appearance of hyperpolarizing GABA_A_R currents is determined by the phosphorylation status of KCC2, a process that facilitates its membrane trafficking and activity^16,17^. Deficits in KCC2 expression levels and activity have been detailed in patient and animal models of epilepsy^18,19^. Furthermore, we, and others, have demonstrated that KCC2 loss of function is strongly correlated with cognitive impairment (in Fragile X syndrome and Rett syndrome) and the development of pharmaco-resistant seizures that are insensitive to GABA_A_R-positive allosteric modulators such as benzodiazepines^20–24^.

The current understanding of whether KCC2 expression, phosphorylation and activity change during development in *Cdkl5* KO pups remains unclear. Furthermore, it is crucial to investigate whether enhancing KCC2 activity during postnatal development, when neural circuits are maturing and the transition from excitation to inhibition occurs in immature neurons, could potentially normalize behavioral and cognitive deficits in *Cdkl5* KO mice. To address this issue, we have developed a novel small-molecule activator, OV350, which potentiates KCC2 activity and effectively terminates pharmaco-resistant seizures in wild-type (WT) mice^25^. Here, we demonstrated the effects of ablating CDKL5 on the phosphorylation of KCC2 and its impact on the development of pharmaco-resistant seizures and cognitive and behavioral deficits. We observed that the pharmacological activation of KCC2 during postnatal development is an important time period for an intervention to improve adult sociability and cognition. We also showed that KCC2 activity during postnatal development reduces adult baseline electroencephalography (EEG) power and restores the ability of diazepam (DZ) to terminate intractable status epilepticus (SE) in *Cdkl5* KO mice.

## Materials and methods

### Study design

This study tested whether early intervention with the KCC2 activator, OV350, during development would correct the phenotypes seen in adult *Cdkl5* KO mice. We selected three phenotypes to test, which correspond to clinical phenotypes observed in CDD patients, namely, seizure/epileptic spasms, spatial memory impairments and behavioral deficiencies. All experiments included mice of both sexes and were conducted across multiple litters, with litters randomized to receive either vehicle (veh) or OV350 treatment. See Table 1 for the key resources.

**Table 1:** Key resources.

Name	Company	Catalog number
Animals		
C57BL/6J	The Jackson Laboratory	000664
6.129(FVB)-Cdkl5tm1.1Joez/J	The Jackson Laboratory	021967
Equipment, chemicals and reagents		
OV350 (KCC2 activator)	Ovid Therapeutics	
Kainic acid	Caymon	58002-62-3
Buprenorphine SR		
DZ	Torics	2805/50
Captisol (SBE-b-CD)	LigandPharmaceuticals	RC-0C7-020
Dimethyl sulfoxide	Sigma	D8418
Mouse KCC2	NeuroMab	N1/12
Mouse actin	Sigma	A1978
Rabbit β-tubulin	Cell Signaling	2128
Rabbit KCC2 pS940	Phosphosolutions	p1551-940
Rabbit KCC2 pT1007	Phosphosolutions	p1551-1007
Rabbit KCC2 PT906	Phosphosolutions	p1551-906
Mouse Cdkl5, clone 8F3.1	Sigma	MABS1132
Protease inhibitor	Roche	11836170001
Phosphatase inhibitor	Roche	4906837001
Protein G Dynabeads	Thermo Fisher	10004D
Bradford assay	Bio-Rad	5000001
Bovine serum albumin	Sigma	A7030
Stereotaxic apparatus	Kopf	
Isothesia (isoflurane)	Henry Schein	SKU 029405
Charcoal filter, VetEquip	Harvard Apparatus	
Paraformaldehyde	Electron Microscopy Sciences	15174-S
EEG/EMG headmounts	Pinnacle Technology	8201
Software		
ImageJ	Schneider et al.^58^	https://imagej.nih.gov/ij/
GraphPad Prism	Prism	
LabChart 8.1	ADInstruments	https://www.adinstruments.com
Ethovision	Noldus	https://www.noldus.com/ethovision

### Animals

The TUFTS University Institutional Animal Care and Use Committee approved all animal use. The animals were housed in temperature-controlled rooms on a 12-h day/night cycle. We purchased the *Cdkl5* KO mice from The Jackson Laboratory (strain 021967) and used a mix of male and female *Cdkl5* KO mice for our experiments. Our study design is focused on evaluating the therapeutic efficacy of KCC2 activation during a specific developmental window (postnatal days (p)10–21) within the Cdkl5^−/−^ genotype. Therefore, we crossed homozygous females and hemizygous males to generate CDD mice (Table 1).

### Drug preparation

OV350 was formulated with 6.25% DMSO and 93.75% (v/v) of 50% (w/v) captisol, and the veh was 6.25% DMSO and 93.75% (v/v) of 50% (w/v) captisol. Mice were injected with 50 mg/kg OV350, a dose which has previously been shown to reach a brain concentration of 676 nM within 4 h and is maintained for 8 h^25^.

### Immunoblotting

Sodium dodecyl sulfate–polyacrylamide gel electrophoresis (SDS–PAGE) was carried out as previously described^26,27^. Detailed methods are provided in the Supplementary Information.

### Plasma membrane isolation

Plasma membranes were isolated as previously described^26,27^. Briefly, rapidly dissected cortical/hippocampal tissues were collected from seven 8–12-week-old male and female mice for each genotype in a starting buffer^28^. Detailed methods are provided in the Supplementary Information.

### Immunoprecipitation

Protein G Dynabeads (Thermo Fisher) were washed and incubated overnight at 4 °C with KCC2 antibody or nonimmune mouse IgG. The beads were washed and crosslinked with dimethyl pimelimidate dissolved in triethanolamine for 30 min at room temperature. The beads were then eluted using nondenaturing soft elution buffer for BN–PAGE as outlined previously^27,29^.

### Protein analysis by LC–MS/MS

Quantitative label-free proteomic analysis was conducted following the previously described method^26,27^. Gel bands of interest were excised and cut into 1-mm^3^ pieces. Subsequently, the peptides were extracted from gel bands, dried and stored at 4 °C before reconstitution in HPLC solvent. The samples were then loaded onto a nano-scale reverse-phase HPLC capillary column. The peptides were detected, isolated and fragmented to produce a tandem mass spectrum of specific fragment ions for each peptide.

### Peptide/protein searches

The MS data in its raw form was processed as previously described^26,27^. Peptide sequences were identified by matching protein or translated nucleotide database sequences with the obtained fragmentation pattern using MSGF+^26,27^. Detailed methods are provided in the Supplementary Information.

### Phosphopeptide proteomic analysis

To measure the impact of CDKL5 removal on global KCC2 phosphorylation, we used proteomics to measure the amount of phosphorylated peptides and unphosphorylated peptides and generate ratios of phosphorylation abundance for each known KCC2 phosphosite, as previously described^26,27^. Three experimental replicates were performed and a *t*-test was used to compare the proteomic data between the two genotypes. Detailed methods are provided in the Supplementary Information.

### Whole-cell patch-clamp recordings

Whole-cell recordings from prefrontal cortex (PFC) neurons were performed in cortical coronal slices (350 μm) from p14–p21 mice. Recording electrodes (5–6 MΩ resistance) contained the following (in mM): 115 K-gluconate, 30 KCl, 10 HEPES, 1 MgCl_2_, 2 Na-ATP and 0.4 Na-GTP, pH 7.4. Slices were continuously perfused with oxygenated artificial cerebrospinal fluid-containing kynurenic acid (3 mM) and bumetanide (10 μM). A picospritzer pipette containing muscimol (5 μM), a GABA_A_R agonist, was used to activate GABA_A_R-mediated currents in cells held at voltages between −90 and −10 mV. Peak amplitude responses of the muscimol-activated current were plotted for each holding voltage and data were fitted by linear regression analysis. The reversal potential of GABA responses (*E*_GABA_) was obtained from the *x*-intercept value of the fit. Voltages were corrected offline with a liquid junction potential value of 13.2 mV. To measure the contribution of KCC2 activity to *E*_GABA_, measurements of *E*_GABA_ were taken before and after a 5-min exposure to VU0463271 (10 μM), a selective inhibitor of KCC2.

### Behavioral spasms

Neonatal pups, aged p10–p21, were removed from their dam and placed in a container on a heating pad for daily sessions lasting 30 min. Behavioral videos were analyzed for spontaneous high-amplitude spastic movements, low-amplitude movements, time spent on their sides and walking. Low-amplitude spasm-like events were categorized as muscle twitches and sudden tail movement between *Cdkl5* KO and WT pups. Analyses were conducted blinded to the treatment and gender, and interrater reliability procedures were considered during the study. High-amplitude spastic movements were characterized by rapid extensions and flexions, involving two to four limbs, spine curving and stumbling, following previously criteria established^30,31^.

### EEG surgeries and recording

Adult mice (8–9-weeks old) were subjected to EEG surgeries as previously described^32^. After the surgery, the mice recovered for 7 days in their home cages before experimentation. On the day of recording, the mice were connected to the pre-amps for recording. First, a 2-h-long baseline recording was obtained and then the mice received a 20 mg/kg kainate (KA) injection (intraperitoneal (i.p.)). Two hours later, the mice were administered with a single dose of 5 mg/kg DZ (i.p.) and were recorded for another hour to assess the effectiveness of postnatal OV350 treatment in adult *Cdkl5* KO mice.

### EEG analysis

To assess the potential impact of OV350 treatment on baseline EEG power, a 20-min silent period was analyzed, during which no muscular movement was detected based on the EMG channel. Mice received a single dose of 50 mg/kg OV350 or veh control for 12 consecutive days during the postnatal development period from postnatal day 10 to 21. To evaluate the effectiveness of OV350 in restoring the efficacy of DZ, a 40-min EEG signal was analyzed and compared to the veh group.

To identify and score seizures and SE, EEG recordings were examined to determine the onset of the first seizure and instances of SE^33^. To investigate the effectiveness of OV350 in preventing the onset of DZ-resistant SE, we compared the EEG epochs of 40 min following DZ administration across all three groups of mice.

### Behavior

For all behavioral tests, WT mice were administered a veh only, while CDD mice were given injections of either a veh or OV350. Animals were randomly assigned to each group and all behavioral analyses were done while blinded to genotype. Interrater reliability procedures were considered during the study. After each experiment, the equipment was sanitized after each mouse using 70% ethanol, followed by Clidox. Both male and female mice were utilized for all experiments.

### Barnes maze assay

Mice aged between 10 and 12 weeks were used for the assay. The maze used was a circular platform with a 1.5 m diameter containing 40 holes along the perimeter, each with a 2.5 cm diameter. An escape tunnel was positioned under one of these holes. Mice used natural spatial cues in the room to locate the escape hole under the maze. The test procedure involved placing the mice in the center of the maze. Afterward, the cage was lifted and the mice were given 3 min to explore the maze and find the escape hole. This test was repeated thrice daily with a 30-min interval between trials. The average duration for each day was considered for analysis. This protocol was followed for four consecutive days.

To assess short-term memory, the escape hole was removed on the fifth day and the mice were given 5 min to explore the arena. The time spent at each hole was measured and put into 45° bins, with each containing five holes. These bins were organized around the perimeter of the maze in a clockwise direction, with 0° representing the goal hole and the adjacent holes. The same assessment was repeated on day 12 after a week of no exposure to the maze for long-term memory. An overhead camera and Ethovision software were used to track the time spent in each area of the arena.

### Three-chamber social interaction assay

At 13 weeks of age, mice were tested in a three-chamber setup, with each chamber measuring 40 cm × 40 cm. The test mice were allowed to explore the arena for 5 min for habituation. After this, an unfamiliar male or female mouse (8 weeks old) was placed under one of the cages and a dummy mouse was placed under the second cage, and the test mice were allowed to explore the arena for 10 min. The time spent in the chamber with the familiar versus the dummy mouse was also calculated. An overhead camera and Ethovision software were used to detect time spent in each region of the arena.

### Statistical analysis

All data are presented as the mean ± s.e.m. The Shapiro–Wilk test was performed to find the normal distribution of datasets. Biochemistry data were analyzed using the Mann–Whitney test or *t*-test. Electrophysiology data were analyzed using a one-way analysis of variance (ANOVA) followed by Tukey’s multiple comparison. Infantile spasm data were analyzed using the Mann–Whitney test or *t*-test. Baseline EEG data were analyzed using the Mann–Whitney test or *t*-test. Seizure data were analyzed using a two-way ANOVA followed by Tukey’s multiple comparison. Behavioral data were analyzed using a two-way ANOVA to compare genotypes followed by Tukey’s multiple comparison, and a repeated measures ANOVA followed by Dunnett’s multiple comparison was used to analyze data obtained from individual mice across different trials. The data are presented in Supplementary Tables 1 and2 (including *P* values, test used, means, s.e.m. and the number of animals). *P* values <0.05 are considered statistically significant.

## Results

### CDKL5 loss is associated with aberrant KCC2 phosphorylation and expression

**Fig. 1:**
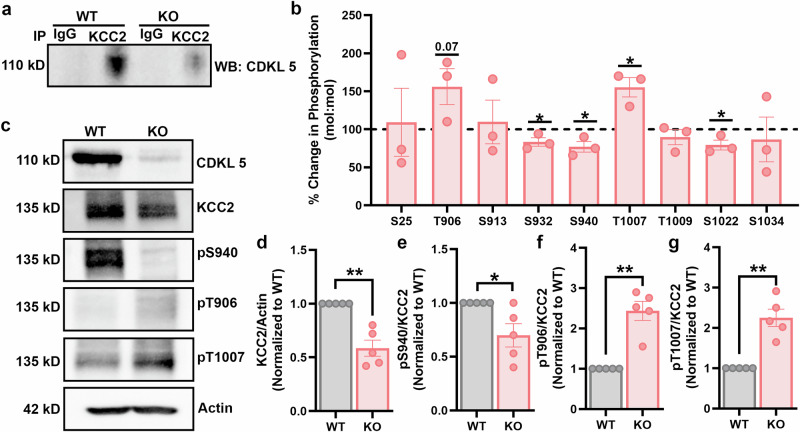
CDKL5 is strongly associated with the KCC2 complex and loss of *Cdkl5* modifies KCC2 phosphorylation. **a** Immunoprecipitation of the KCC2 complex in plasma membrane preparations shows a strong association of CDKL5 with KCC2 in the WT mice when blotted with Cdkl5 antibody. CDKL5 is reduced in the KCC2 complex in *Cdkl5* KO mice. **b** Spectra searches were performed on LC–MS/MS data obtained from purified KCC2 to identify phosphorylated residues. The S932, S940 and S1022 residue phosphorylation was significantly reduced in *Cdkl5* KO mice compared to the WT mice. However, T906 and T1007 phosphorylation of KCC2 is increased in the *Cdkl5* KO mice compared to the WT mice. **c** Forebrain lysates prepared from the WT or *Cdkl5* KO mice were immunoblotted with β-actin, Cdkl5, KCC2 and phospho-specific antibodies against S940 (pS940), T906 (pT906) and T1007 (pT1007). **d** The graph shows a reduction in KCC2 expression in *Cdkl5* KO mice compared to the WT mice. **e** S940 phosphorylation of KCC2 is reduced in *Cdkl5* KO mice compared to the WT mice. **f** T906 phosphorylation of KCC2 is increased in the *Cdkl5* KO mice compared to the WT mice. **g** KCC2 phosphorylation at T1007 is increased in *Cdkl5* KO mice compared to the WT mice. The asterisk indicates the significance of the *Cdkl5* KO mouse compared to WT. See Supplementary Table 1 for the statistical analysis.

To confirm the association between CDKL5 and KCC2, we isolated the cortex and hippocampus and made purified plasma membrane preparations that were subjected to immunoprecipitation with KCC2 or control IgG antibodies. Precipitated material was immunoblotted with the CDKL5 antibody. In WT mice, CDKL5 is associated with KCC2 and is part of the KCC2 complex. CDKL5 was significantly reduced in the *Cdkl5* KO mice (Fig. 1a). Next, we analyzed the phosphorylation status of KCC2 residues in both *Cdkl5* KO and WT mice. Global phosphorylation analysis between the two groups was conducted using LC–MS/MS. Spectral searches identified phosphorylated serine (S) and threonine (T), which are crucial sites for post-translational modifications that influence the trafficking, plasma membrane expression and chloride extrusion capabilities of the KCC2 transporter. We estimated phosphorylation levels on these residues by comparing the quantities of phosphorylated versus dephosphorylated peptides. The S940 site, which is known to positively influence KCC2 activity, exhibited significantly lower phosphorylation levels in *Cdkl5* KO mice compared to WT mice. By contrast, the T906 and T1007 sites, recognized for negatively regulating KCC2 activity, showed significantly higher phosphorylation levels in *Cdkl5* KO mice than in WT mice. Furthermore, we found a notable reduction in phosphorylation on two novel residues, S932 and S1022, in *Cdkl5* KO mice compared to WT mice (Fig. 1b). Cortical and hippocampal lysates prepared from WT or *Cdkl5* KO mice (8–12 weeks old) were immunoblotted with antibodies against β-actin, CDKL5 and KCC2 and phospho-specific antibodies pS940, pT906 and pT1007. Compared to WT mice, there was a significant reduction in KCC2 expression in *Cdkl5* KO mice (Fig. 1c,d). There was a concomitant reduction in the phosphorylation level of KCC2 at the serine 940 site (Fig. 1c,e). By contrast, phosphorylation is increased at T906 (Fig. 1c,f) and T1007 (Fig. 1c,g) sites in *Cdkl5* KO mice. These results demonstrate that loss of CDKL5 results in a KCC2 phosphorylation phenotype that displays the hallmarks of an immature phenotype characterized by dysfunctional KCC2 activity.

### CDKL5 loss orchestrates the alteration in postnatal regulation of KCC2 expression and phosphorylation

**Fig. 2:**
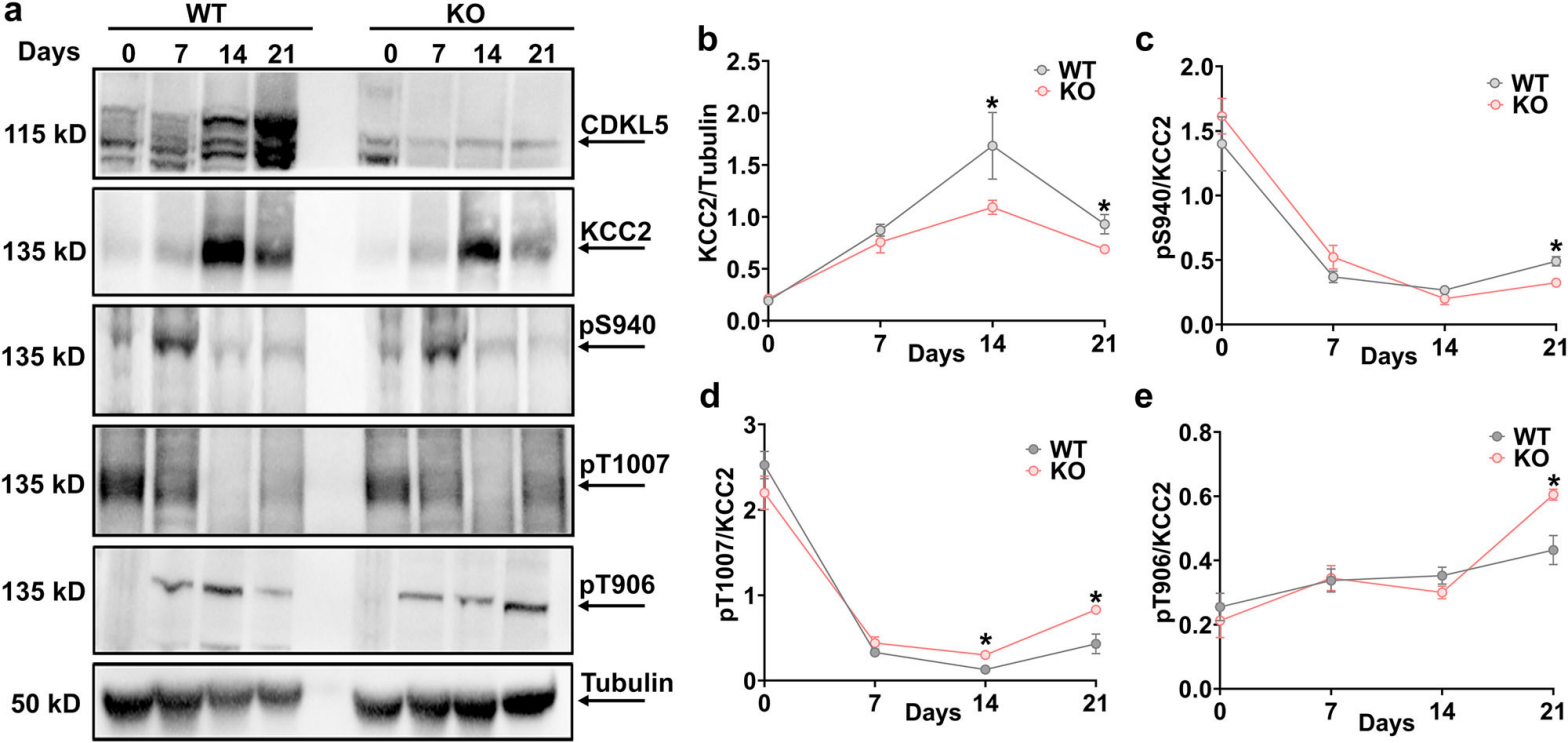
CDKL5 ablation alters KCC2 expression and phosphorylation over time during postnatal development. **a** Western blotting was used to assess developmental changes in KCC2 expression and phosphorylation in WT and *Cdkl5* KO pups at 0, 7, 14 and 21 postnatal days. Whole-brain lysates prepared from WT or *Cdkl5* KO pups were immunoblotted with β-tubulin, CDKL5, KCC2 and phospho-specific antibodies against S940 (pS940), T906 (pT906) and T1007 (pT1007). **b** KCC2 expression progressively increased from p0 to p14 and stabilized by p21 in WT and *Cdkl5* KO pups. However, KCC2 expression from p14 to p21 is significantly reduced in *Cdkl5* KO mice compared to WT pups. **c** Relative to the levels of KCC2 expression, S940 phosphorylation is decreased from p0 to p21 in WT and *Cdkl5* KO pups. S940 phosphorylation was significantly reduced at p21 in *Cdkl5* KO pups compared to WT pups. **d** The graph shows a progressive reduction in T1007 phosphorylation over time in WT pups relative to KCC2 expression. Meanwhile, *Cdkl5* ablation significantly increased phosphorylation at the T1007 site on KCC2 from p14 to p21 compared to WT pups. **e** T906 phosphorylation on KCC2 does not change over time from p0 to p21 during postnatal development in WT and *Cdkl5* KO mice. However, the phosphorylation is significantly increased at the T906 site on KCC2 at p21 in *Cdkl5 KO* pups. The asterisk indicates the significance of the *Cdkl5* KO mouse compared to WT on a particular day. See Supplementary Table 1 for the statistical analysis.

Phosphorylation of KCC2 at residues serine 940 (S940), threonine 1007 (T1007) and threonine 906 (T906) regulates KCC2 function in the adult brain, so we sought to examine the developmental profile of S940, T906 and T1007 phosphorylation. We measured total KCC2 expression and KCC2 S940, T906 and T1007 phosphorylation in whole-brain lysates at p0, p7, p14 and p21 and detected phosphorylation of this residue at each of these time points (Fig. 2a). Total KCC2 expression increased linearly over development until p14, followed by maintenance at this level as the brain neurons further matured by p21 in WT mice, with significantly lower expression at p14 and p21 in *Cdkl5* KO pups compared to WT pups (Fig. 2b). We detected a significant decrease in S940 phosphorylation at p21 between WT and *Cdkl5* KO pups (Fig. 2c). Phosphorylation of KCC2 at threonine residues 906 and 1007 decreases during development in WT mice, which contributes to the developmental upregulation of KCC2 function. Therefore, we also compared the phosphorylation status at these residues between WT and *Cdkl5* KO pups. We observed significant increases in KCC2 phosphorylation at T1007 from p14 onward in *Cdkl5* KO pups compared to WT pups (Fig. 2d). We also detected a significant increase in phosphorylation at T906 at p21 in *Cdkl5* KO pups compared to the WT pups (Fig. 2e). An increase in phosphorylation in these threonine residues during development in *Cdkl5* KO pups suggests a developmental delay in the maturation of KCC2.

These results demonstrate that loss of CDKL5 results in a KCC2 phosphorylation phenotype that leads to decreased KCC2 function, which is strongly correlated with excessive neuronal excitation, seizure-like events, increased seizure susceptibility, and cognitive and behavioral impairments in adult mice^34,35^.

### *Cdkl5* KO pups show a population of neurons that have depolarized *E*_GABA_ values

To examine KCC2 activity in neurons from *Cdkl5* KO mice compared to WT mice, we employed a whole-cell patch-clamp Cl^−^ loading assay^35,36^. By imposing an internal 32 mM Cl^−^ load through the patch pipette, the subsequent measurement of *E*_GABA_ reports the degree to which these neurons were able to extrude a fixed amount of Cl^−^. Without KCC2 activity, the Nernst equation dictates that neurons should have an *E*_GABA_ value of −39 mV when loaded with 32 mM [Cl^−^]_i_. The WT neurons displayed an *E*_GABA_ value of −57.3 ± 2.5 mV (*n* = 7), indicating the presence of a functional Cl^−^ extrusion KCC2. PFC neurons from *Cdkl5* KO mice displayed two populations, one that did not display any action potential at the cell’s resting membrane potential, similar to WT, and another population that displayed action potential firing at rest. The former population had an *E*_GABA_ value of −61.2 ± 2.9 mV (*n* = 7). The latter had a depolarized *E*_GABA_ value of −44.2 ± 3.6 mV (*n* = 4) that was significantly more depolarized compared to WT or the electrically silent *Cdkl5* KO group of neurons (Supplementary Fig. 1a,b). Additional experiments were performed to validate the contribution of KCC2 activity to *E*_GABA_. We measured *E*_GABA_ before and after a 5-min exposure to VU0463271 (10 μM), a selective inhibitor of KCC2. The neurons from the *Cdkl5* KO mice that displayed an increased excitability had a significantly smaller depolarizing shift in *E*_GABA_ (4.1 ± 2.5 mV, *n* = 6) compared to WT (11.1 ± 1.1 mV, *n* = 11) and the less excitable *Cdkl5* KO neurons (14 ± 2 mV, *n* = 9; Supplementary Fig. 1c). This indicates that the population of neurons with depolarized *E*_GABA_ has inadequate functional KCC2 to contend with the Cl^−^ load.

### *Cdkl5* KO pups show intense infantile spasms

Dysfunction or mutations in KCC2 in mouse models and human patients often lead to infantile epileptic spasms^37–39^. Our proteomics data show a significant reduction in the expression of KCC2 and alterations in the phospho-profile of KCC2 during the postnatal developmental period, which may contribute to the infantile spasms in *Cdkl5* KO mice.

Therefore, we next examined whether behavioral spasms occur in *Cdkl5* KO pups during neonatal development. Since the onset of infantile spasms in humans is usually in the first year of life, we quantified behavioral spasms in *Cdkl5* KO and WT littermate pups between p10 and p21. These ages represent a period of significant synapse development and brain growth in the respective species. Infantile spasms were defined as spontaneous high-amplitude, spastic movements, using the criteria developed for assessing infantile spasm-like phenotypes in other rodent models^30,31^.

**Fig. 3:**
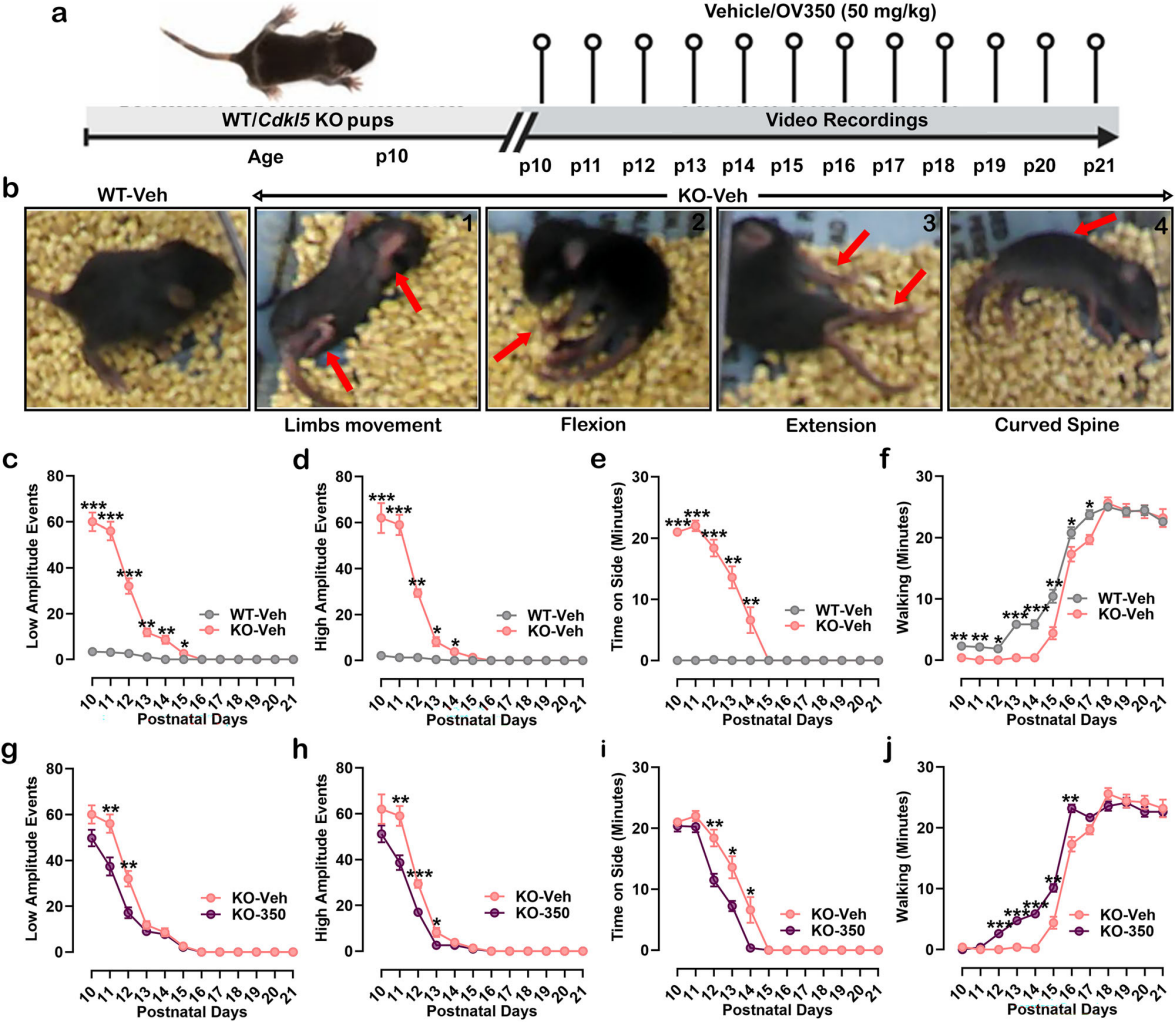
KCC2 activation reduces behavioral spasms in neonatal *Cdkl5* KO pups. **a** The experimental timeline. **b** Representative example of a WT pup and *Cdkl5* KO pups experiencing spontaneous high-amplitude spastic movements, including movement of two to all four limbs (1), rapid full flexion (2), extension of limbs (3) and and spine curving (4). **c** The number of low-amplitude spastic movements was increased in *Cdkl5* KO pups compared to WT pups from postnatal day 10–15, based on scoring of the 30-min video recording of pup behavior. **d** High-amplitude movements were significantly more frequent in *Cdkl5* KO pups from p10 to 14 than in WT pups. **e** *Cdkl5* KO pups spent significantly more time on their sides, indicative of an abnormal behavioral state from p10 to 14 compared to WT pups. **f** *Cdkl5* KO pups spent less time walking than WT pups from p10 to 17. **g** *Cdkl5* KO pups treated with OV350 showed a reduced number of low-amplitude spastic movements compared to veh-treated KO pups on postnatal days 11–12. **h** High-amplitude movements were significantly less in OV350-treated *Cdkl5* KO pups from p11 to 13 compared to veh-treated KO pups. **i** OV350-treated *Cdkl5* KO pups spent significantly less time on their sides than veh-treated KO pups from p12 to 14. **j** *Cdkl5* KO pups treated with OV350 spent more time walking than veh-treated KO pups from p12 to 15. The asterisk indicates the significant difference in infantile spasms and motor activities among different groups of mice. See Supplementary Table 1 for the statistical analysis.

The spasms typically occurred in a series and were separated by periods of low- and high-amplitude spasm-like events and behavioral arrest, often with the mouse remaining on its side (Fig. 3a,b). We first compared low-amplitude spasm-like events, such as muscle twitches and tail movement, between *Cdkl5* KO and WT pups. The *Cdkl5* KO pups exhibited significantly more low-amplitude spasm-like events than WT pups from p10 to p15 (Fig. 3c). No difference in low-amplitude events was observed from p16 to p21 between the two groups of mice. Next, we quantified the quantity of high-amplitude spasm-like events (clusters of rapid, full flexions, extensions, rapid movement of two to four limbs, sudden stumbling and spine curving) between *Cdkl5* KO and WT pups. The number of high-amplitude spasms was significantly higher in *Cdkl5* KO pups than in WT pups from p10 to p14 (Fig. 3d). No difference in high-amplitude events was observed from p15 to p21 between the two groups of mice. In addition, *Cdkl5* KO pups spent more time on their side than WT pups from p10 to p14 (Fig. 3e) but not from p15 to p21. Also, *Cdkl5* KO pups spent significantly less time walking during p10–p17 but started to walk at comparable levels to WT from p18 to p21 (Fig. 3f). Overall, *Cdkl5* KO pups showed a delay in achieving important developmental milestones. These results suggest that infantile spasms in *Cdkl5* KO mice may result from compromised KCC2 activity, potentially leading to improper neuronal circuit formation and contributing to abnormal behavior and developmental delays.

### Potentiating KCC2 activity during the postnatal developmental period reduces infantile spasms in *Cdkl5* KO pups

We have previously demonstrated that enhancing KCC2 activity through genetic animal models or a novel small-molecule activator increases the seizure threshold and terminates ongoing status epilepticus^16,25^. We further investigated the effect of enhancing KCC2 activity during the critical development phase (p10–21) on infantile spasms. To test this hypothesis, we administered a daily dose of the KCC2 activator (OV350, 50 mg/kg i.p.) to *Cdkl5* KO pups from p10 to p21, in comparison to age-matched, veh-treated *Cdkl5* KO pups (Fig. 3a). Our observations revealed that OV350-treated *Cdkl5* KO pups had significantly fewer low-amplitude spasm-like events than their veh-treated counterparts from p11 to p12 (Fig. 3g). In addition, KCC2 activation with OV350 decreased the occurrence of high-amplitude spasm-like events in *Cdkl5* KO pups from p11 to p13 (Fig. 3h). The OV350-treated *Cdkl5* KO pups also demonstrated significantly less time spent on their side than veh-treated KO pups from p12 to p14 (Fig. 3i) and more time walking than their veh-treated KO pups from p12 to p15 (Fig. 3j). These findings indicate that enhancing KCC2 activity during development can lessen infantile spasms in *Cdkl5* KO pups and potentially support the formation of normal neuronal circuits during this critical time in development, which may improve deficits in neuronal excitability, sociability and spatial memory.

### Adult *Cdkl5* KO mice exhibit increased baseline EEG power, which is normalized by OV350 treatment during the postnatal developmental period

**Fig. 4:**
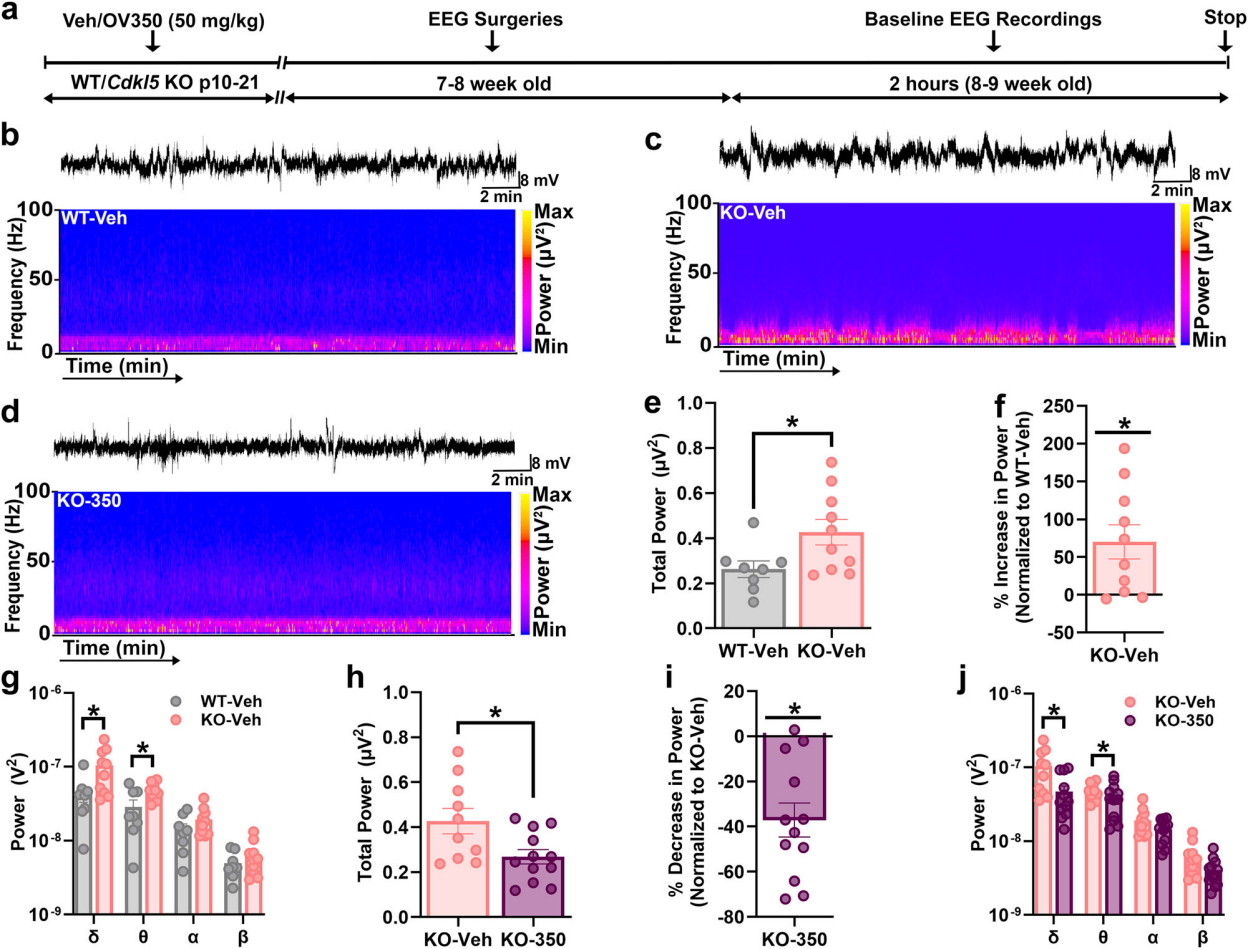
KCC2 activator reduces the baseline EEG power in *Cdkl5* KO mice. **a** The line diagram shows the experimental timeline. Two hours of baseline EEG recordings were performed after veh/OV350 (50 mg/kg i.p., arrow) administration during the postnatal development period (p10–p21). **b** Representative EEG trace and its spectrogram show the power distribution across different frequency bands from a veh-treated WT mouse. **c** The representative EEG trace is shown above, along with the power spectra from a veh-treated *Cdkl5* KO mouse. **d** The representative EEG trace is shown above, along with the power spectra from an OV350-treated *Cdkl5* KO mouse. **e** Baseline EEG recordings from WT-veh and KO-veh groups of mice were subjected to FFT, and a spectral plot is shown for frequencies between 0 and 100 Hz. The total EEG power was compared between the two groups of mice. *Cdkl5* KO-veh mice had a higher baseline EEG power than WT-veh mice. **f** The graph shows a percentage increase in EEG power. **g** The EEG power is significantly increased across delta and theta frequency bands in *Cdkl5* KO-veh mice compared to the WT-veh mice. **h** Mice treated with OV350 significantly reduced the EEG power compared to the veh-treated *Cdkl5* KO mice. **i** The graph shows a percentage decrease in EEG power. **j** The EEG power is significantly decreased across the delta and theta frequency bands in *Cdkl5* KO-350 mice compared to the KO-veh mice. The asterisk indicates the significant difference in EEG power between different groups of mice. See Supplementary Table 1 for the statistical analysis.

The postnatal change in KCC2 phosphorylation regulates the developmental GABA switch. Disruption to the timing of the switch (as would be expected in *Cdkl5* KO mice with an immature KCC2 phosphorylation signature) will have long-lasting consequences for many developing brain circuits, altering their long-term excitability^40^^,41^. Here, we used EEG recordings to measure the baseline EEG signal in adult WT and *Cdkl5* KO mice. We further examined the consequences of enhancing KCC2 activity during this critical developmental period on baseline EEG measurements. To address this hypothesis, we administered a daily dose of the KCC2 activator (OV350, 50 mg/kg i.p.) to *Cdkl5* KO pups from p10 to p21, compared to veh-treated, age-matched, WT and *Cdkl5* KO pups. After 4 weeks, EEG surgeries were performed. In the following week after recovering from the surgeries, a 2-h-long baseline recording was conducted to examine the difference in baseline EEG power between the WT-vehi, *Cdkl5* KO-veh and *Cdkl5* KO-OV350 mice (Fig. 4a). A 20-min-long EEG epoch recording was compared between all three groups. To quantify the difference, recordings were subjected to fast Fourier transformation (FFT) to convert the EEG signals from the time domain into the frequency domain, generating a power spectral density plot for frequencies between 0 and 100 Hz (Fig. 4b–d). Similar to the human CDD patients^42^, we observed that the total baseline EEG power of *Cdkl5* KO-veh mice was significantly higher than that of WT-veh mice (Fig. 4e,f). To further find that which EEG frequency bands were contributing to an increase in total power, we also assessed the possible differences in the EEG frequency bands (delta, 0–4 Hz; theta, 4–8 Hz; alpha, 8–13 Hz; and beta, 13–30 Hz) and again, similar to the human CDD patients^42^ we observed a significant increase in power in the delta and theta frequency bands (Fig. 4g). Next, we compared whether KCC2 activation using OV350 during postnatal development reduces baseline EEG power in *Cdkl5* KO mice. We observed that the total baseline EEG power of *Cdkl5* KO-350 mice was significantly reduced compared to the KO-veh mice (Fig. 4h,i). Again, when we compared the power distribution across OV350 and veh-treated Cdkl5 KO mice, we observed that the *Cdkl5* KO mice treated with OV350 while they were infants showed a significant reduction in power across the delta and theta frequency bands compared to the veh-treated Cdkl5 KO mice (Fig. 4j). These results suggest that the *Cdkl5* KO mice may be more prone to seizures due to the higher baseline EEG power. Therefore, we compared the susceptibility to seizures and SE between the two groups of mice.

### *Cdkl5* KO mice are more susceptible to KA-induced seizures and status epilepticus

**Fig. 5:**
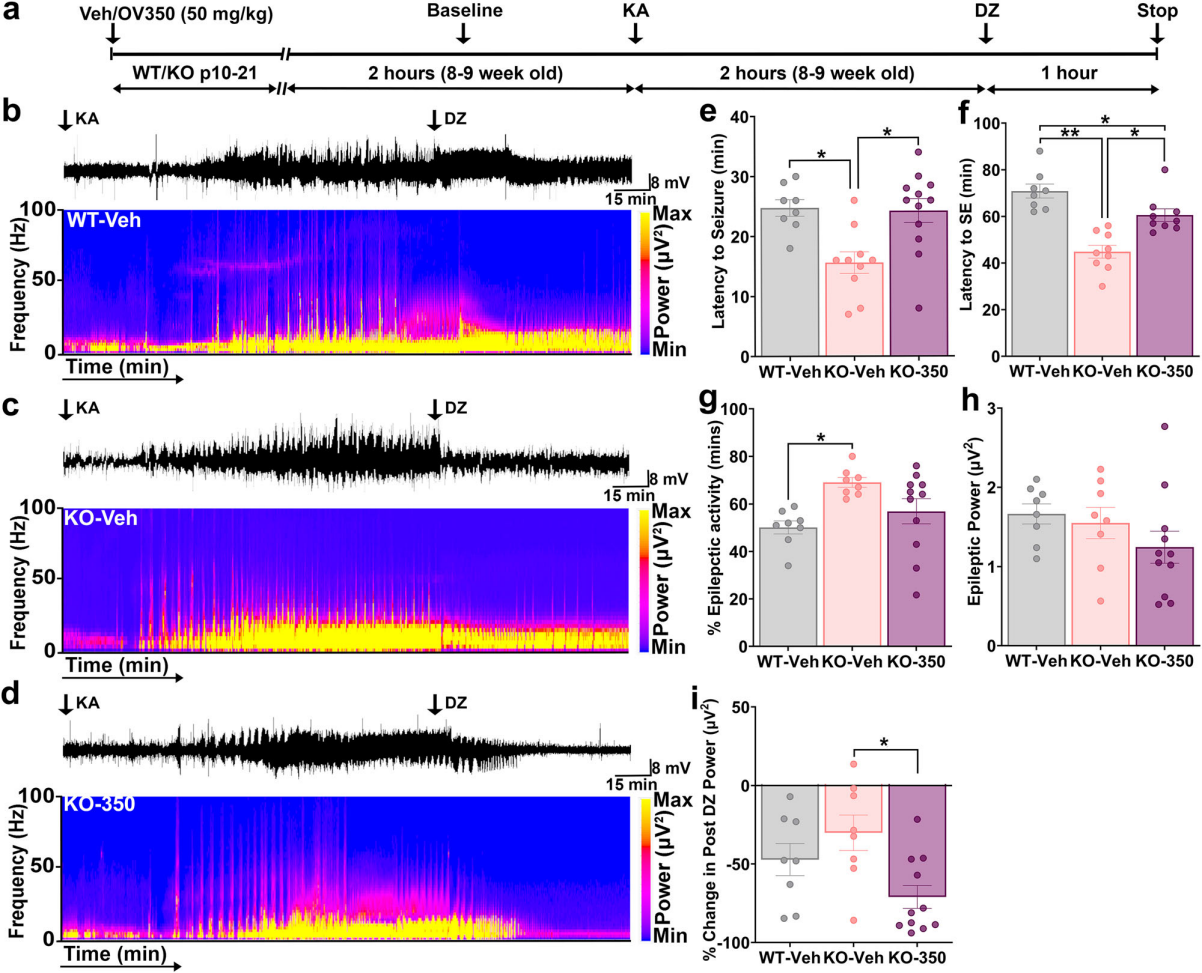
KCC2 activator increases the susceptibility to KA-induced seizures and SE in *Cdkl5* KO mice. **a** The experimental timeline shows that WT pups were administered with the veh and *Cdkl5* KO pups received either veh or OV350 (50 mg/kg i.p.) from p10 to p21 during the postnatal development period. Four weeks later, these mice underwent EEG surgeries. One week following the surgeries, on the day of the experiment, a 2 h baseline recording was conducted. Next, mice received a single injection of KA (20 mg/kg i.p., first arrow) to induce seizures and status epilepticus. Two hours after KA injection, mice were dosed (i.p.) with 5 mg/kg DZ, and EEG recordings were extended for 1 h. **b** Representative EEG trace and its spectrogram show power distribution across different frequency bands from a veh-treated WT mouse. **c** Representative EEG trace and spectrogram show power distribution across different frequency bands from a veh-treated *Cdkl5* KO mouse. **d** A representative EEG trace is shown above in the power spectra of an OV350-treated *Cdkl5* KO mouse. **e** The graph shows increased latency to the first seizure in *Cdkl5* KO mice. The asterisk indicates the significant difference in latency to the first seizure among different groups of mice. **f** OV350-treated *Cdkl5* KO mice took more time to get into KA-induced SE. The asterisk indicates the significant difference in latency to the SE among different groups of mice. **g** Veh-treated WT mice spent less time in epileptic events than the veh-treated *Cdkl5* KO mice, and OV350 treatment did not reduce time spent in epileptic activity in the *Cdkl5* KO mice. The asterisk indicates the significant difference in time spent in epileptic activity among different groups of mice. **h** EEG recordings of the epileptic activity from the veh- and OV350-treated mice were subjected to FFT, and a spectral plot is shown for frequencies between 0 and 100 Hz. The total EEG power was compared between all three groups of mice. OV350 treatment did not alter the EEG power in *Cdkl5* KO mice. **i** EEG recordings of the post-DZ treatment period from all three groups of mice were subjected to FFT, and a spectral plot is shown for frequencies between 0 and 100 Hz. The total EEG power was compared. Mice treated with OV350 had significantly reduced EEG power compared to the veh-treated mice. The asterisk indicates the significant difference in change in post-DZ power among different groups of mice. See Supplementary Table 1 for the statistical analysis.

Since KCC2 phosphorylation influences seizure severity, *Cdkl5* KO mice with altered KCC2 phosphorylation would be predicted to have increased duration of epileptiform activity, a decreased latency to the first epileptiform event and a decreased latency to onset of SE compared to WT mice. To address this hypothesis, we compared the susceptibility of KA-induced seizures in WT and *Cdkl5* KO mice. A single dose of 20 mg/kg KA was administered, with recordings continuing for 2 h. After that, all three groups of mice received a saturating concentration of DZ (5 mg/kg i.p.), after which recordings were extended for an additional 1 h (Fig. 5a–d).

This model was chosen because of the similarities with patients having drug-resistant seizures as KA-induced seizures become refractory to benzodiazepines within minutes. The *Cdkl5* KO mice took less time to have their first seizure (Fig. 5e) and developed SE faster than WT mice (Fig. 5f). In addition, the *Cdkl5* KO mice spent more time in epileptic activity than the WT mice (Fig. 5g). However, we did not observe a difference in the EEG power of total epileptic activity (Fig. 5h). In both WT and *Cdkl5* KO mice, the benzodiazepine, DZ, failed to suppress SE, as demonstrated by the lack of change in EEG power before and after DZ treatment (Fig. 5i).

### Targeting KCC2 activity in infant *Cdkl5* KO mice reduces seizure susceptibility and restores DZ efficacy in adult mice

We next explored if these seizure susceptibility issues observed in adult *Cdkl5* KO mice could be negated by enhancing KCC2 function during development. Adult *Cdkl5* KO mice that received OV350 treatment between p10 and p21 had a significantly increased latency to the first KA-induced seizure compared to veh-treated *Cdkl5* KO mice (Fig. 5e). These mice also took a significantly longer time to develop status epilepticus than the veh-treated *Cdkl5* KO mice (Fig. 5f). However, KCC2 activation did not reduce the time spent in epileptic activity and epileptic power in *Cdkl5* KO mice (Fig. 5g,h). Consistent with previously published studies using the KA model, DZ did not modify EEG power in veh-treated mice. By contrast, in *Cdkl5* KO mice treated with OV350 (50 mg/kg i.p.) between p10 and p21, DZ significantly reduced EEG power in adult mice (Fig. 5i).

### OV350 treatment during postnatal development improves sociability in adult *Cdkl5* KO mice

**Fig. 6:**
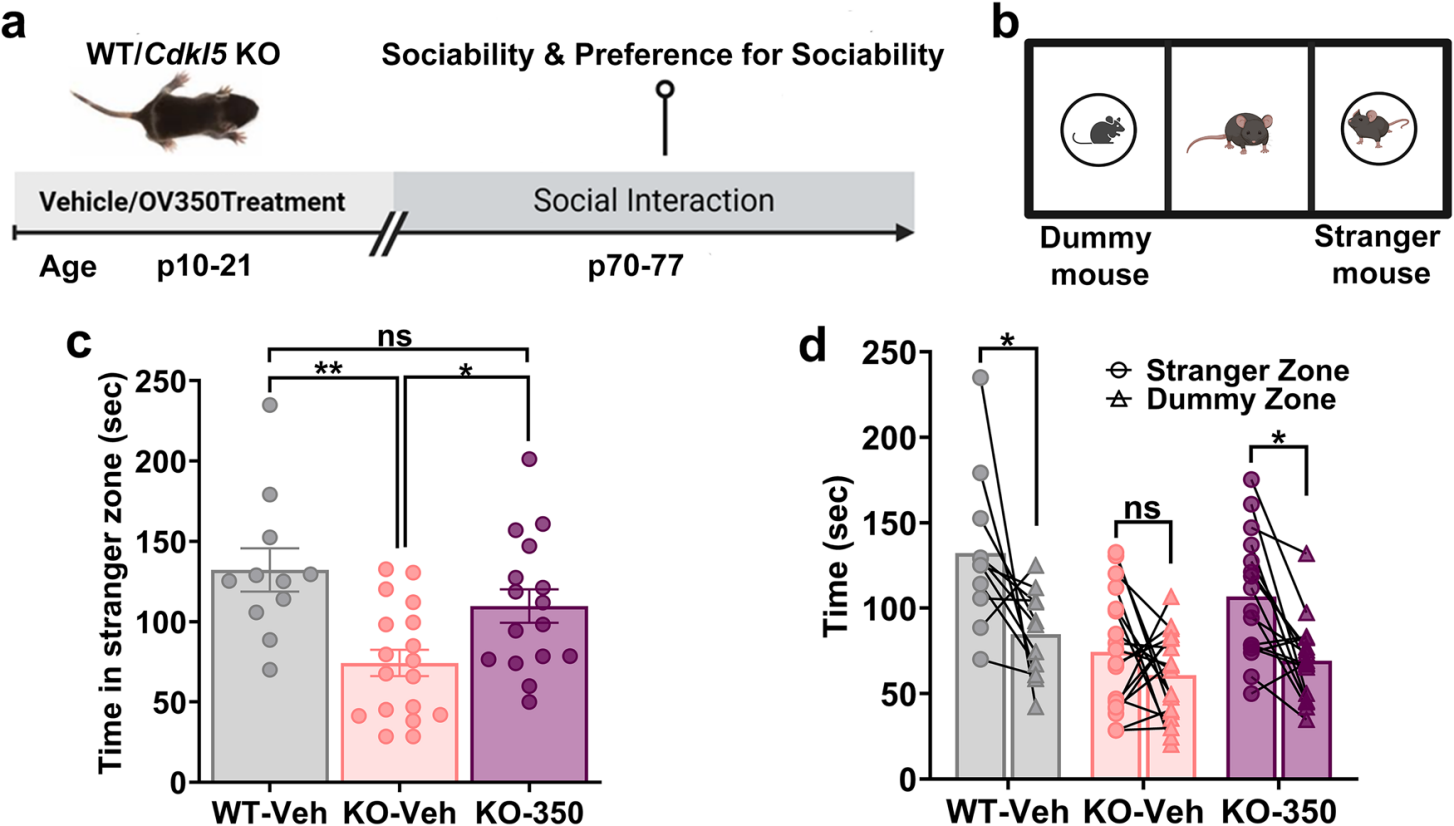
KCC2 activation during development improves sociability in *Cdkl5* KO mice. **a** The experimental timeline. The WT pups were administered a single dose of veh daily from p10 to p21. The *Cdkl5* KO pups received a single dose of either vehor OV350 (50 mg/kg i.p.) daily from p10 to p21. We used a three-chamber social interaction assay to assess sociability and preference for social familiarity in the *Cdkl5* KO mice treated with or without OV350. **b** A diagram of the sociability assay. Mice were allowed to explore either an unfamiliar mouse or a dummy mouse. **c** WT mice spent more time interacting with the stranger mouse than veh-treated *Cdkl5* KO mice, and OV350-treated *Cdkl5* KO mice spent more time interacting with the stranger mouse than veh-treated mice. The asterisk indicates the significance of the time spent with the stranger mouse. **d** The graph shows that both WT and OV350-treated *Cdkl5* KO mice preferred interacting with the mouse versus the dummy mouse. The asterisk indicates the significance of the time spent with the dummy and the stranger mouse. See Supplementary Table 1 for the statistical analysis.

The *Cdkl5* KO mice exhibit autistic-like behavioral abnormalities and perform poorly in social interaction tasks. Deficits in KCC2 expression and phosphorylation also promote autism-like behavior in mice^14,43^. However, increasing KCC2 activity during development improves sociability in mice^16^. Therefore, we investigated the impact of enhancing KCC2 activity using OV350 (50 mg/kg i.p.) during development (p10–p21) on social behavior in adult *Cdkl5* KO mice (10–11 weeks) (Fig. 6a). We hypothesized that the abnormal social behavior in *Cdkl5* KO mice is linked to altered KCC2 phosphorylation and reduced KCC2 activity, and potentiating KCC2 activity during development would rescue sociability in *Cdkl5* KO mice. Sociability was assessed using a three-chamber social interaction test (Fig. 6b). First, sociability was examined by measuring time spent interacting with an unfamiliar (stranger) mouse. Consistent with the literature*, Cdkl5* KO-veh mice spent significantly less time interacting with the stranger mouse than the WT-veh mice did, indicating that the *Cdkl5*-deficient mice have a reduced motivation for social interaction. However, the OV350-treated *Cdkl5* KO mice spent more time interacting with the stranger mouse compared to the time *Cdkl5* KO-veh mice spent, suggesting that potentiating KCC2 activity during development alleviates deficits in sociability in *Cdkl5-*deficient mice (Fig. 6c). Next, we measured how much time mice spend with a live mouse compared to a dummy mouse. *Cdkl5* KO-veh-treated mice spent almost the same time with live and dummy mice. However, *Cdkl5* KO-OV350 mice, like WT mice, spent more time with a live mouse (Fig. 6d). This finding suggests that KCC2 activation during development in *Cdkl5* KO mice increases their preference for sociability as adult mice.

### Administering OV350 during postnatal development improves spatial learning in *Cdkl5* KO mice

**Fig. 7:**
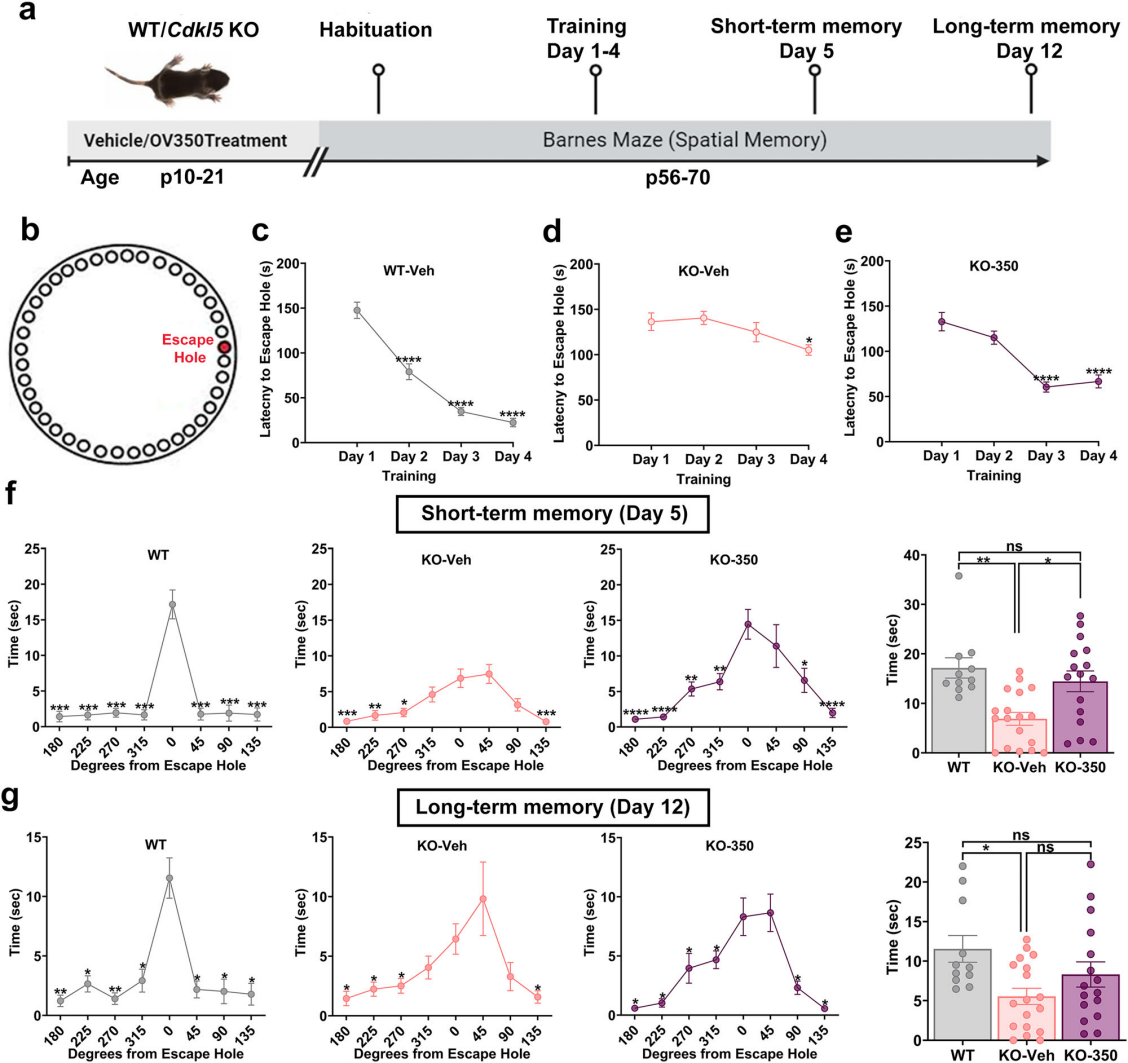
KCC2 activation during the postnatal development period improves the rate of spatial learning and spatial memory retention in *Cdkl5* KO mice. **a** The experimental timeline. The *Cdkl5* KO pups received a single dose of either veh or OV350 (50 mg/kg i.p.) daily from p10 to p21. WT pups were administered a single dose of veh daily from p10 to p21. We used a Barnes maze assay to assess the rate of spatial learning in the WT-veh and *Cdkl5* KO mice treated with or without OV350. **b** A cartoon illustration of the Barnes maze. **c** Latency to enter the goal hole was measured on days 1–4; the learning rate (day when there is a significant reduction in latency to goal compared to day 1) was comparable in WT mice. The asterisk indicates the significant difference in latency to the escape hole with reference to day 1. **d** The learning rate was comparable in veh-treated *Cdkl5* KO mice from day 4. The asterisk indicates the significant difference in latency to the escape hole with reference to day 1. **e** The learning rate was comparable in OV350-treated *Cdkl5* KO mice from day 3. The asterisk indicates the significant difference in latency to the escape hole with reference to day 1. **f** Spatial memory was assessed using a Barnes maze assay. After 4 days of training, the time spent at each hole was measured on day 5 and day 12 upon removal of the escape tunnel and data were binned into 45° groups. WT-veh mice spent more time in the goal area on day 5. Veh-treated *Cdkl5* KO mice did not perform as well as the WT mice. OV350-treated (50 mg/kg i.p.) *Cdkl5* KO mice performed considerably better. WT-veh and OV 350-treated *Cdkl5* KO mice spent more time at the goal zone than the veh-treated *Cdkl5* KO mice. **g** WT mice show enhanced specificity of the spatial memory on day 12 compared to the veh- and OV350-treated *Cdkl5* KO mice. The WT mice spent more time at the goal than the other two groups of mice. *Indicates significant difference to time spent in the 0° region. See Supplementary Table 1 for the statistical analysis.

Intellectual disabilities are a core feature of CDD and compromised KCC2 phosphorylation also impacts learning and memory. We performed a Barnes maze assay to examine spatial learning and memory in the *Cdkl5* KO mice. We treated *Cdkl5* KO pups with OV350 (50 mg/kg i.p.) or veh during development (p10–p21), and WT mice received veh injections only. We then examined the beneficial effects of increasing KCC2 activity during development on spatial memory in adult *Cdkl5* KO mice (8–10 weeks old) (Fig. 7a,b). To assess the impact of treatment on learning, we recorded the latencies to enter the escape hole over 4 days of learning. *Cdkl5* KO-veh mice performed poorly in escape latencies compared to the WT mice over the 4-day learning period, while the OV350-treated mice performed better than the veh-treated *Cdkl5* KO mice but not as well as the WT mice (Supplementary Fig. 2). The WT mice showed a significant improvement over their day one escape latencies by day 2 (Fig. 7c). The *Cdkl5* KO-veh showed an improvement in escape latencies by training day 4 (Fig. 7d). By contrast, the *Cdkl5* KO-350 mice showed a significant improvement in escape latencies by training day 3 (Fig. 7e). This suggests that the rate of spatial learning was mildly improved in the OV350-treated *Cdkl5* KO mice.

### Potentiating KCC2 activity during postnatal development impacts spatial memory in *Cdkl5* KO mice

Memory assessments were conducted over increasing periods after the learning portion of the Barnes maze assay. We assessed short-term and long-term memory on day 5 and day 12, respectively. To examine memory retention, we removed the escape chamber to measure the time spent in the space where the escape hole was initially located. Time spent at the escape hole differed between WT and *Cdkl5* KO-veh mice on day 5, suggesting that the short-term memory was significantly impaired in the *Cdkl5* KO-veh mice. Interestingly, OV350-treated *Cdkl5* KO mice spent more time investigating the escape hole region than other regions in the maze on day 5 (Fig. 7f). By day 12, the *Cdkl5* KO-veh mice could not differentiate between the escape and nonescape regions, but the WT mice showed long-term memory retention and spent more time in the escape hole region. While the OV350-treated *Cdkl5* KO mice demonstrated a preference for the escape hole region compared to the nonescape hole region, they did not spend a significant time at the escape hole (Fig. 7g), suggesting that increasing KCC2 function during development improves short-term spatial memory retention as adults but has limited impact on long-term memory.

## Discussion

Our findings suggest that direct activation of KCC2 during postnatal development in *Cdkl5* KO mice normalizes baseline EEG power and enhances the effectiveness of DZ in terminating drug-induced SE. In addition, we observed that KCC2 activation during development alleviates sociability and cognitive deficits in *Cdkl5* KO mice.

KCC2 functions by extruding intracellular chloride anions and is essential for the ontogenetic switch of GABA_A_-mediated responses from depolarizing to hyperpolarizing^14^. The expression and function of KCC2 play a crucial role in regulating neuronal excitability, and disruptions in KCC2 have been associated with the onset of infantile epilepsy^44^. Our findings indicate a significant decrease in KCC2 expression during postnatal development, as well as in adult *Cdkl5* KO mice. This reduction in KCC2 expression may result from the activation of the BDNF–TrkB pathway in the *Cdkl5* KO mice^45^. Notably, BDNF levels are elevated in *Cdkl5* KO mice, which is known to negatively impact KCC2 expression^46^. It is well established that phosphorylation of KCC2 at the serine 940 site (pS940) is critical for stabilizing KCC2 on the neuronal membrane surface, enabling the extrusion of Cl^−^ and maintaining KCC2 activity^17^. Consistent with an earlier report^13^, we observed a significant reduction in phosphorylation at the S940 site on KCC2 in the whole-brain and forebrain lysates from *Cdkl5* KO mice. In addition, we noted a significant increase in phosphorylation at the threonine 1007 and 906 sites on KCC2 (pKCC2–T1007), which inhibits its transport function^35^. These findings are in line with the literature, indicating that the serine and threonine kinomes are altered in *Cdkl5* KO mice^10^. This alteration may result from the inactivation of the mTOR signaling pathway^10^, which is essential for the phosphorylation of KCC2^47^. Phosphorylation at these sites is necessary for the switch of GABA_A_-mediated excitation to inhibition during the second week of postnatal development. Our findings of altered phosphorylation suggest a potential delay in this critical developmental switch, which may contribute to hyperexcitability and infantile seizures in both human patients and animal models of CDD.

In the PFC of *Cdkl5* KO mice, two populations of neurons were found, one that had an *E*_GABA_ comparable to neurons from WT mice and a second population that had depolarized *E*_GABA_ that was close to the predicted *E*_GABA_ value for the Cl^−^ loading conditions of the experiment, indicating that there was no active Cl^−^ exclusion by KCC2 in that particular population. Owing to the limited circuit activity in ex vivo brain slices, the demand on KCC2 is low, hence the comparable *E*_GABA_ values of WT and some *Cdkl5* KO neurons. We have previously described in KCC2 S940A mice that KCC2 distribution and levels of Cl^–^ extrusion were comparable to WT^34^. It was only when the brain slice was challenged with glutamate that KCC2 activity in S940A mice was unable to contend with the subsequent rise in [Cl^−^]_i_ resulting from increased circuit and neuronal activity. Similarly, we predict that due to the KCC2 phosphorylation changes observed in *Cdkl5* KO mice, there will be an increase in neuronal activity (particularly an increase in GABAergic interneuron activity), and KCC2 in *Cdkl5* KO neurons will not be able to react to the rise in [Cl^−^]_i_. We have demonstrated the occurrence of epileptic-like high-amplitude spasms in *Cdkl5* KO mice between the ages p10 and p14, which resolved thereafter. *E*_GABA_ values were collected from p14–p21 mice; future studies will examine *E*_GABA_ in younger mice to explore the possibility that a greater proportion of neurons in the age range of high-amplitude spasms (p10–p14) will have more depolarized *E*_GABA_ values.

Neuronal and circuit hyperexcitability has been noted in EEG recordings from patients with CDD^42^ and EEG and brain slice recordings from conditional *Cdkl5* knockout mice^48^, as well as multielectrode recordings of neurons from organoids from patients with CDD^49^. To investigate this phenomenon, we compared baseline EEG power in *Cdkl5* KO mice to that of WT mice. Our analysis revealed a significant increase in baseline EEG power among the *Cdkl5* KO mice. This is in line with EEG studies conducted on human patients^42^, where we observed elevated EEG power across the delta and theta frequency bands. These findings suggest that quantitative EEG measurements may serve as reliable biomarkers for diagnosing CDD and for the effective development of new treatments. Furthermore, we found that administering a KCC2 activator during postnatal development is sufficient to normalize EEG power levels in adult *Cdkl5* KO mice. Currently, there is a lack of validated biomarkers to assess brain function and clinical severity in individuals with CDD, which complicates the objective evaluation of emerging treatments. Therefore, we propose the use of quantitative EEG parameters as objective measures of brain function and disease severity in future clinical trials for CDD.

In rodent studies, SE induced by KA is resistant to termination by DZ, modeling patients experiencing drug-resistant seizures^32,50–52^. Our research indicates that *Cdkl5* KO mice exhibit a shorter time to first seizure and develop DZ-resistant SE more quickly than WT mice. Furthermore, the *Cdkl5* KO mice tend to spend more time engaged in epileptic activity, potentially due to their elevated basal EEG power. Notably, we discovered that repeated administration of OV350 during postnatal development was sufficient in delaying the onset of DZ-resistant seizures and SE in adult *Cdkl5* KO mice.

Major autistic-like phenotypes, such as social and communication deficits, are hallmark characteristics of CDD^8,24,53,54^. In line with existing literature, our findings indicate that *Cdkl5* KO mice performed significantly worse in social interaction tasks than their WT counterparts. We observed an alleviation of sociability deficits when we enhanced KCC2 activity in *Cdkl5* KO mice during postnatal development using OV350. However, activating KCC2 in adulthood did not lead to a reduction in these social deficits. This underscores the importance of early intervention during postnatal development as this period is crucial for forming neural circuits and the accumulation of KCC2, which facilitates the transition from excitation to inhibition in immature neurons.

Mice that lack CDKL5 exhibit several key characteristics associated with the neurological disorder, including impairments in hippocampal-dependent memory and deficits in motor coordination. While both young adult and middle-aged *Cdkl5* KO mice demonstrate significant learning and memory deficits, they do not present motor impairments at an early age. Consistent with these observations, our studies revealed impairments in spatial learning and memory retention among *Cdkl5* KO mice. We found that enhancing KCC2 activity during the postnatal development of these *Cdkl5* KO mice led to long-term improvements in hippocampus-dependent spatial learning and memory retention. As a result, these mice performed better in learning the Barnes maze task and exhibited improved short-term memory. This aligns with our previous research, which indicated that the constitutive activation of KCC2 through the development of a transgenic mouse line enhanced spatial learning and memory. Recently, a study using CDD mouse models also found that re-expression of Cdkl5, while the mice were young (from 6 weeks), reverses many CDD-related phenotypes. Therefore, our findings reaffirm the notion that early interventions may be the most effective strategy to reduce cognitive deficits in *Cdkl5* KO mice.

Several limitations warrant consideration before applying these findings to patient populations. First, we investigated only a single dose of OV350 (50 mg/kg), previously shown to reverse DZ-resistant seizures in adult mice^25^. Infants may need lower doses to achieve clinical efficacy. Second, we treated for 12 days during development (p10–p21), it remains to be seen whether a shorter time course of treatment would be equally effective. Third, the effect of OV350 on spontaneous seizures remains unknown. Owing to the absence of spontaneous seizures in older mice, we used KA-induced seizures, which respond to acute OV350 treatment^25^. We have observed infantile spasms in young *Cdkl5* KO mice, and a previous study observed seizures recorded by EEG in p12 mice^13^. Future experiments are needed to determine the developmental stage at which these seizure events start and how long they last; this will determine the length and time course of treatment. Fourth, we focused on studying whether KCC2 activation via OV350 application during a specific developmental window (p10–p21) could reverse cognitive, social and epileptic phenotypes in CDD mice. In future experiments, an important control group would be WT pups treated with OV350 during the same developmental window (p10–p21). Including this control would allow us to assess whether KCC2 activation also influences behavior in adult WT mice. If WT mice showed no changes, it would have strengthened our findings that OV350 specifically rescues disease phenotypes in CDD mice.

Previous studies have implicated impaired chloride homeostasis in Rett syndrome, Fragile X syndrome and other DEEs, which contribute to network hyperexcitability^23,55,56^. Pharmacological enhancement of the KCC2 gene expression has been shown to have disease-modifying effects in Rett syndrome^24^. Here, we report that in addition to changes in expression, KCC2 phosphorylation is also altered in *Cdkl5* KO mice during development in such a manner as to impair KCC2 activity. It remains to be determined whether similar alterations in KCC2 phosphorylation underlie the impaired transporter activity observed in Rett and Fragile X syndromes. Our data indicate that reduced KCC2 expression and phosphorylation changes in CDD present a critical challenge during the postnatal development period with therapeutic implications that differ from those observed in other DEEs. Specifically, we show that pharmacological activation of KCC2 during a defined postnatal window (p10–p21) not only suppresses epileptic spasms but also rescues cognitive and social behaviors in adult CDD mice. This suggests that early intervention targeting chloride extrusion can recalibrate circuit function before irreversible developmental deficits are established. While these results are promising, extrapolation to human CDD remains speculative and requires further studies. Human patients and the CDD mouse model have differences in developmental timing, as p10 mice are comparable to a 1-year-old child^57^, as well as variations in drug metabolism, safety profiles and patient variability that must be carefully addressed in future translational efforts. Overall, this study demonstrates that enhancing KCC2 function during infancy is a potential therapy for CDD and other developmental and epileptic encephalopathies.

## Supplementary information

Supplementary Information
