# Supplementary material for: KCC2 activation during postnatal development alleviates long-term deficits in CDKL5-deficient mice
Source: Exp Mol Med. 2026 Feb 19;58(2):591–604. doi: 10.1038/s12276-026-01670-x (PMC12992657; doi:10.1038/s12276-026-01670-x)
Supplement: Supplementary file 1 — Supplementary Information [file 12276_2026_1670_MOESM1_ESM.pdf]

Methods:

Immunoblotting

Sodium dodecyl sulfate-polyacrylamide gel electrophoresis (SDS-PAGE) was carried out as previously described [26, 27]. Samples were diluted in 2x sample buffer, and 30 μg of protein was loaded onto a 7% polyacrylamide gel. Next, proteins were transferred onto a nitrocellulose membrane, blocked, and probed with primary antibodies. The membranes were washed and incubated for 1 h at room temperature with HRP-conjugated secondary antibodies. Protein bands were visualized with Pierce ECL and imaged using a ChemiDoc MP (Bio-Rad). Band intensity was compared to beta-actin and beta-tubulin as loading controls.

Plasma membrane isolation

Plasma membranes (PM) were isolated as previously described[26, 27]. Briefly, rapidly dissected cortical/hippocampal tissues from seven 8–12 weeks old male and female mice for each genotype were collected in a starting buffer [28]. Tissues from 7 animals were pooled and homogenized in isolation buffer supplemented with protease and phosphatase inhibitors. The homogenates were subjected to serial differential centrifugation to isolate the purified PM fraction. The PM fraction was solubilized in Triton lysis buffer supplemented with protease and phosphatase inhibitors.

Immunoprecipitation

Protein G Dynabeads (Thermo Fisher) were washed and incubated overnight at 4°C with KCC2 antibody or non-immune mouse IgG. The beads were washed and crosslinked with dimethyl pimelimidate dissolved in triethanolamine for 30 mins at room temperature. After that, the beads were resuspended in solubilized PM fractions and incubated overnight at 4°C. The beads were then eluted using non-denaturing soft elution buffer for BN-PAGE as outlined previously [27, 29].

Densitometry

For Western blot analysis, bands from raw images were analyzed using densitometry. Biological replicates were run on the same gels for comparison, and each band's area under the curve was calculated. The average signal for each treatment group was calculated based on the protein expression levels.

Protein Analysis by LC-MS/MS

The quantitative label-free proteomic analysis was conducted following the previously described method [26, 30]. Gel bands of interest were excised and cut into 1 mm^3 pieces. Subsequently, in-gel trypsin digestion was carried out, followed by washing and dehydration of the gel pieces with acetonitrile. The gel pieces were then rehydrated with a solution containing trypsin and incubated before being subjected to ammonium bicarbonate solution and overnight incubation. The peptides were extracted, dried, and stored at 4 °C before reconstitution in HPLC solvent. The samples were then loaded onto a nano-scale reverse-phase HPLC capillary column. The eluting peptides were subjected to Nanospray ionization and then entered into an LTQ Orbitrap Velos Pro ion-trap mass spectrometer for analysis. The peptides were detected, isolated, and fragmented to produce a tandem mass spectrum of specific fragment ions for each peptide.

Peptide/protein searches

The MS data in its raw form was processed as previously described [26, 30]. Peptide sequences were identified by matching protein or translated nucleotide database sequences with the obtained fragmentation pattern using MSGF+ [26, 30]. We performed searches on raw .mzXML files against the UniProt mouse reference proteome and the Thermo list of common contaminants. The search parameters encompassed settings for high-resolution Orbitrap mass spectrometers, tryptic

digestion, no restriction on enzyme missed cleavages, a 20 ppm precursor mass tolerance, charge states ranging from +2 to +5, minimum and maximum peptide lengths of 6–40 amino acids, and a fixed modification of standard amino acids with carbamidomethyl (C + 57). Peptide identification was evaluated using MSGF + Q- (PSM-level target-decoy approach) and E- (expected number of peptides in a random database) scores. These scores were utilized for quality assurance during the initial protein screening process for associated proteins and phospho-modified peptide screening.

Phosphopeptide proteomic analysis

In order to measure the impact of CDKL5 removal on global KCC2 phosphorylation, we used proteomics to measure the amount of phosphorylated peptides and unphosphorylated peptides and generate ratios of phosphorylation abundance for each known KCC2 phosphosite as previously described[26, 30]. Briefly, raw.mzXML files were compared to the UniProt mouse reference proteome and the Thermo list of common contaminants, to detect peptides for KCC2 with or without mass shifts associated with phosphorylation (79.97 Da). The amounts of phosphorylated and unphosphorylated peptides for each known site were compared to generate ratios of phosphorylation abundance. These were used to look for shifts in phosphorylation abundance at each known high-confidence KCC2 phosphorylation site.

Supplementary Figures

Supplementary Figure 1

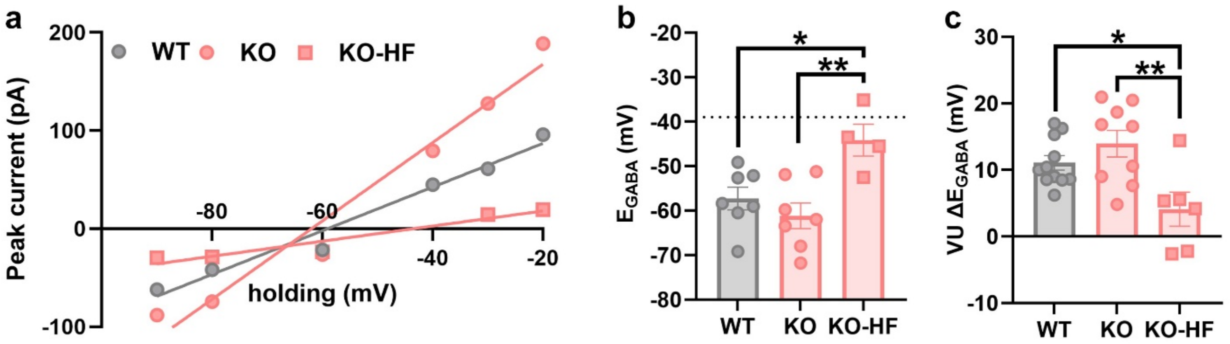

Supplementary Fig. 1. CDKL5 ablation creates a population of neurons in PFC that shows depolarized E_{GABA} values. (**a**) Current–voltage (I–V) plots elicited by rapid application of muscimol to PFC neurons in slices from WT (grey circles), *Cdkl5* KO (pink circles), and *Cdkl5* KO high firing (pink squares) mice (p14-21) loaded with 32-mM Cl^- at differing voltages. (**b**) Summary of E_{GABA} values in neurons from PFC WT and KO mice. Neurons that had a high basal action potential firing had a more depolarized E_{GABA} compared to WT or low-firing KO neurons. High firing KO neurons had E_{GABA} values near to −39 mV (dashed line), the predicted reversal potential when there is no KCC2 activity. (**c**) Shifts in E_{GABA} are shown for PFC neurons from WT and KO mice, calculated as a change in E_{GABA} (ΔE_{GABA}) following a 5-min exposure to the KCC2 inhibitor VU0463271 (VU). In all panels, data represent mean ± SEM. See Supplementary Table 2 for statistical analysis.

Supplementary Figure 2

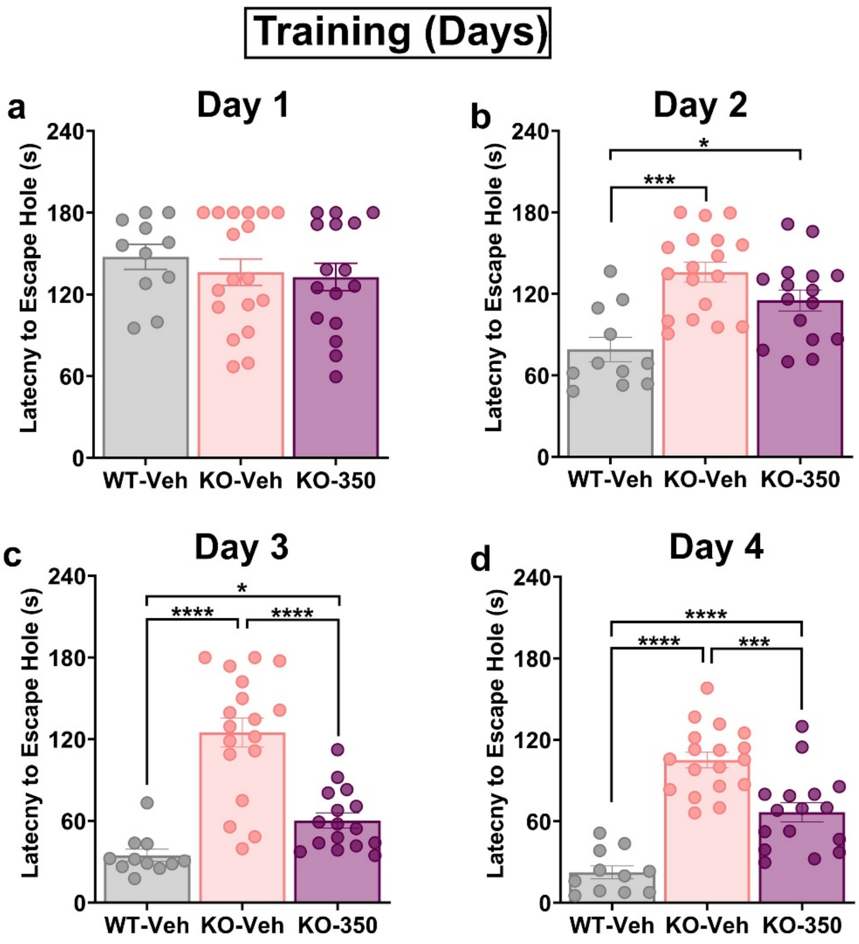

Supplementary Fig.2. Potentiating KCC2 activity during development improves spatial learning in *Cdkl5* KO mice.

Mice were exposed to the maze for four consecutive days (three training sessions each day), learning to associate the location of an escape hole using training room spatial cues**.** (**a**) The latency to enter the escape hole was similar among the three treatment groups. (**b**) The WT mice took less time to enter the escape hole than the vehicle and OV350-treated (50 mg/kg *i.p.*) *Cdkl5*-KO mice on day 2. (**c**) On day 3, OV350-treated *Cdkl5* KO mice showed significant improvement in spatial learning compared to vehicle-treated *Cdkl5* KO mice, but didn't bring it to the WT level. (**d**) On day 4, OV350-treated *Cdkl5* KO mice showed further improvement in spatial learning compared to vehicle-treated *Cdkl5* KO mice, but didn't bring it to the WT level. The asterisk indicates the significant difference in latency to the escape hole. See Supplementary Table 1 for statistical analysis.

Supplementary Table 1: Statistical Analysis

**Fig. 1b**									
**Quantitative Measurements**	**S25**	**T906**	**S913**	**S932**	**S940**	**T1007**	**T1009**	**S1022**	**S1034**
t-test: *p-value*	0.84	0.07	0.75	0.04	0.03	0.01	0.35	0.03	0.67
Mean (% Change in Phosphorylation)	WT:100 KO: 109	WT:100 KO: 156	WT:100 KO: 109	WT:100 KO: 83.33	WT:100 KO: 77	WT:100 KO: 155.3	WT:100 KO: 89.67	WT:100 KO: 79.33	WT:100 KO: 86.67
Standard Error of Mean	WT:0 KO: 44.7	WT:0 KO: 23.58	WT:0 KO: 28.7	WT:0 KO: 5.6	WT:0 KO: 7.0	WT:0 KO: 12.7	WT:0 KO: 9.9	WT:0 KO: 6.4	WT:0 KO: 29.38
n (Replicates) and N (animals)	WT: 3 (21) KO: 3 (21)	WT: 3 (21) KO: 3 (21)	WT: 3 (21) KO: 3 (21)	WT: 3 (21) KO: 3 (21)	WT: 3 (21) KO: 3 (21)	WT: 3 (21) KO: 3 (21)	WT: 3 (21) KO: 3 (21)	WT: 3 (21) KO: 3 (21)	WT: 3 (21) KO: 3 (21)

**Fig. 1d-g**				
**Quantitative Measurements**	**KCC2**	**pS940**	**pT1007**	**pT906**
Mann-Whitney test: *p-value*	0.0079	0.04	0.0079	0.0079
Mean (Integrated density)	WT: 1.0 KO: 0.5860	WT: 1.0 KO: 0.6980	WT: 1.0 KO: 2.250	WT: 1.0 KO: 2.434
Standard Error of Mean	WT: 0.0 KO: 0.07481	WT: 0.0 KO: 0.1083	WT: 0.0 KO: 0.2142	WT: 0.0 KO: 0.2360
Median & Difference	WT: 1.0 KO: 0.54 =-0.46	WT: 1.0 KO: 0.7 =-0.3	WT: 1.0 KO: 2.17 =1.17	WT: 1.0 KO: 2.530 =1.530
N (animals)	WT: 5 KO: 5	WT: 5 KO: 5	WT: 5 KO: 5	WT: 5 KO: 5

**Fig. 2b: KCC2/Tubulin**				
**Quantitative Measurements**	**P0**	**P7**	**P14**	**P21**
t-test: *p-value*	0.11	0.36	0.01	0.04
Mean (Integrated density)	WT: 0.1925 KO: 0.2125	WT: 0.8725 KO: 0.7575	WT: 0.210 KO: 1.093	WT: 0.9300 KO: 0.6900
Standard Error of Mean	WT: 0.008 KO: 0.002	WT: 0.0566 KO: 0.1038	WT: 0.2103 KO: 0.067	WT: 0.093 KO: 0.029
N (animals)	WT: 4 KO: 4	WT: 4 KO: 4	WT: 4 KO: 4	WT: 4 KO: 4
**Fig. 2c: pS940/KCC2**				

Quantitative Measurements	P0	P7	P14	P21
t-test: *p-value*	0.42	0.1825	0.2434	0.01
Mean (Integrated density)	WT: 1.4<br>KO: 1.615	WT: 0.37<br>KO: 0.5225	WT: 0.2675<br>KO: 0.200	WT: 0.490<br>KO: 0.325
Standard Error of Mean	WT: 0.2092<br>KO: 0.1374	WT: 0.044<br>KO: 0.091	WT: 0.025<br>KO: 0.045	WT: 0.035<br>KO: 0.029
N (animals)	WT: 4<br>KO: 4	WT: 4<br>KO: 4	WT: 4<br>KO: 4	WT: 4<br>KO: 4
**Fig. 2d: pT1007/KCC2**				
**Quantitative Measurements**	**P0**	**P7**	**P14**	**P21**
t-test: *p-value*	0.246	0.223	0.02	0.02
Mean (Integrated density)	WT: 2.525<br>KO: 2.200	WT: 0.3300<br>KO: 0.4400	WT: 0.1295<br>KO: 0.300	WT: 0.4300<br>KO: 0.8300
Standard Error of Mean	WT: 0.1601<br>KO: 0.1958	WT: 0.040<br>KO: 0.070	WT: 0.0068<br>KO: 0.0195	WT: 0.1145<br>KO: 0.0408
N (animals)	WT: 4<br>KO: 4	WT: 4<br>KO: 4	WT: 4<br>KO: 4	WT: 4<br>KO: 4
**Fig. 2e: pT906/KCC2**				
**Quantitative Measurements**	**P0**	**P7**	**P14**	**P21**
t-test: *p-value*	0.5553	0.8916	0.1628	0.01
Mean (Integrated density)	WT: 0.2550<br>KO: 0.2125	WT: 0.3375<br>KO: 0.3450	WT: 0.3525<br>KO: 0.300	WT: 0.3175<br>KO: 0.8160
Standard Error of Mean	WT: 0.0427<br>KO: 0.0529	WT: 0.0356<br>KO: 0.0388	WT: 0.02658<br>KO: 0.0195	WT: 0.1174<br>KO: 0.0410
N (animals)	WT: 4<br>KO: 4	WT: 4<br>KO: 4	WT: 4<br>KO: 4	WT: 4<br>KO: 4

Fig. 3c: Low amplitude spastic events

Quantitative Measurements	P10	P11	P12	P13	P14	P15
t-test: *p-value*	0.0001	0.0002	0.0008	0.003	0.008	0.01
Mean	WT: 3.4<br>KO: 60	WT: 3.4<br>KO: 60	WT: 3.4<br>KO: 60	WT: 3.4<br>KO: 60	WT: 3.4<br>KO: 60	WT: 3.4<br>KO: 60
Standard Error of Mean	WT: 0.4<br>KO: 4.0	WT: 0.4<br>KO: 4.0	WT: 0.64<br>KO: 3.39	WT: 0.26<br>KO: 1.71	WT: 0.0<br>KO: 1.8	WT: 0.0<br>KO: 1.03
N (animals)	WT: 7<br>KO: 5	WT: 7<br>KO: 5	WT: 7<br>KO: 5	WT: 7<br>KO: 5	WT: 7<br>KO: 5	WT: 7<br>KO: 5
**Fig. 3d: High amplitude spastic events**						
**Quantitative Measurements**	**P10**	**P11**	**P12**	**P13**	**P14**	**P15**
t-test: *p-value*	0.0007	0.0002	0.001	0.01	0.02	0.296

Mean	WT: 2.14<br>KO: 62.0	WT: 1.2<br>KO: 59	WT: 1.2<br>KO: 29.4	WT: 0.42<br>KO: 8.2	WT: 0.0<br>KO: 3.86	WT: 0.0<br>KO: 1.4
Standard Error of Mean	WT: 0.4<br>KO: 6.4	WT: 0.2<br>KO: 4.3	WT: 0.2<br>KO: 1.69	WT: 0.2<br>KO: 1.98	WT: 0.0<br>KO: 1.06	WT: 0.0<br>KO: 1.4
N (animals)	WT: 7<br>KO: 5	WT: 7<br>KO: 5	WT: 7<br>KO: 5	WT: 7<br>KO: 5	WT: 7<br>KO: 5	WT: 7<br>KO: 5

Fig. 3e: Time on Side

**Quantitative Measurements**	**P10**	**P11**	**P12**	**P13**	**P14**	**P15**
t-test: *p-value*	0.0001	0.0001	0.0002	0.001	0.002	ns
Mean	WT: 0<br>KO: 21.0	WT: 0<br>KO: 22	WT: 0.14<br>KO: 18.4	WT: 0.0<br>KO: 13.6	WT: 0.0<br>KO: 6.6	WT: 0.0<br>KO: 0.0
Standard Error of Mean	WT: 0.0<br>KO: 0.54	WT: 0.0<br>KO: 0.8	WT: 0.14<br>KO: 18.4	WT: 0.0<br>KO: 13.6	WT: 0.0<br>KO: 2.1	WT: 0.0<br>KO: 0.0
N (animals)	WT: 7<br>KO: 5	WT: 7<br>KO: 5	WT: 7<br>KO: 5	WT: 7<br>KO: 5	WT: 7<br>KO: 5	WT: 7<br>KO: 5

Fig. 3f: Walking

**Quantitative Measurements**	**P10**	**P11**	**P12**	**P13**	**P14**	**P15**	**P16**	**P17**
t-test: *p-value*	0.003	0.007	0.02	0.0001	0.0001	0.002	0.04	0.02
Mean	WT: 2.2<br>KO: 0.4	WT: 2.1<br>KO: 0.0	WT: 1.8<br>KO: 0.0	WT: 5.8<br>KO: 0.4	WT: 5.8<br>KO: 0.4	WT: 10.3<br>KO: 4.4	WT: 20.7<br>KO: 17.3	WT: 23.7<br>KO: 19.7
Standard Error of Mean	WT: 0.4<br>KO: 0.2	WT: 0.3<br>KO: 0.0	WT: 0.59<br>KO: 0.0	WT: 0.5<br>KO: 0.4	WT: 0.7<br>KO: 0.2	WT: 1.06<br>KO: 0.97	WT: 0.8<br>KO: 1.2	WT: 0.7<br>KO: 0.76
N (animals)	WT: 7<br>KO: 5	WT: 7<br>KO: 5	WT: 7<br>KO: 5	WT: 7<br>KO: 5	WT: 7<br>KO: 5	WT: 7<br>KO: 5	WT: 7<br>KO: 5	WT: 7<br>KO: 5

Fig. 3g: Low amplitude spastic events

**Quantitative Measurements**	**P10**	**P11**	**P12**	**P13**	**P14**	**P15**
t-test: *p-value*	0.08	0.007	0.003	0.11	0.68	0.7
Mean	KO-Veh: 60<br>KO-350: 49.7	KO-Veh: 56<br>KO-350: 37.3	KO-Veh: 32<br>KO-350: 17.1	KO-Veh: 11.8<br>KO-350: 9.0	KO-Veh: 8.6<br>KO-350: 7.75	KO-Veh: 2.6<br>KO-350: 2.12
Standard Error of Mean	KO-Veh: 4<br>KO-350: 3.6	KO-Veh: 4.0<br>KO-350: 3.91	KO-Veh: 3.3<br>KO-350: 2.4	KO-Veh: 1.7<br>KO-350: 0.84	KO-Veh: 1.8<br>KO-350: 0.75	KO-Veh: 1.03<br>KO-350: 0.58
N (animals)	KO-Veh: 5<br>KO-350: 8	KO-Veh: 5	KO-Veh: 5<br>KO-350: 8	KO-Veh: 5<br>KO-350: 8	KO-Veh: 5<br>KO-350: 8	KO-Veh: 5<br>KO-350: 8

		KO-350: 8				
**Fig. 3h: High amplitude spastic events**						
**Quantitative Measurements**	**P10**	**P11**	**P12**	**P13**	**P14**	**P15**
t-test: *p-value*	0.18	0.005	0.003	0.04	0.38	0.76
Mean	KO-Veh: 62 KO-350: 51	KO-Veh: 59 KO-350: 38.63	KO-Veh: 29.4 KO-350: 17	KO-Veh: 8.2 KO-350: 2.6	KO-Veh: 3.8 KO-350: 2.6	KO-Veh: 1.4 KO-350: 1.0
Standard Error of Mean	KO-Veh: 6.4 KO-350: 3.6	KO-Veh: 4.3 KO-350: 3.1	KO-Veh: 1.6 KO-350: 1.2	KO-Veh: 1.9 KO-350: 0.4	KO-Veh: 1.0 KO-350: 0.6	KO-Veh: 1.16 KO-350: 0.42
N (animals)	KO-Veh: 5 KO-350: 8	KO-Veh: 5 KO-350: 8	KO-Veh: 5 KO-350: 8	KO-Veh: 5 KO-350: 8	KO-Veh: 5 KO-350: 8	KO-Veh: 5 KO-350: 8
**Fig. 3i: Time on side**						
**Quantitative Measurements**	**P10**	**P11**	**P12**	**P13**	**P14**	**P15**
t-test: *p-value*	0.56	0.18	0.003	0.01	0.04	ns
Mean	KO-Veh: 21.0 KO-350: 20.0	KO-Veh: 22 KO-350: 20.25	KO-Veh: 18.4 KO-350: 11.5	KO-Veh: 13.6 KO-350: 7.25	KO-Veh: 6.6 KO-350: 0.3	KO-Veh: 0 KO-350: 0
Standard Error of Mean	KO-Veh: 0.54 KO-350: 0.9	KO-Veh: 0.83 KO-350: 0.92	KO-Veh: 1.36 KO-350: 1.05	KO-Veh: 1.8 KO-350: 0.83	KO-Veh: 2.1 KO-350: 0.26	KO-Veh: 0 KO-350: 0
N (animals)	KO-Veh: 5 KO-350: 8	KO-Veh: 5 KO-350: 8	KO-Veh: 5 KO-350: 8	KO-Veh: 5 KO-350: 8	KO-Veh: 5 KO-350: 8	KO-Veh: 5 KO-350: 8

Fig. 3j: Walking

**Quantitative Measurements**	**P10**	**P11**	**P12**	**P13**	**P14**	**P15**	**P16**	**P17**
t-test: *p-value*	ns	0.007	0.02	0.0001	0.0001	0.002	0.04	0.02
Mean	KO-Veh: 0 KO-350: 0	KO-Veh: 0 KO-350: 0.6	KO-Veh: 0 KO-350: 2.6	KO-Veh: 0.4 KO-350: 4.7	KO-Veh: 0.2 KO-350: 5.8	KO-Veh: 4.4 KO-350: 10.1	KO-Veh: 17.3 KO-350: 23.1	KO-Veh: 19.7 KO-350: 21.6

Standard Error of Mean	KO-Veh: 0<br>KO-350: 0	KO-Veh: 0<br>KO-350: 0.3	KO-Veh: 0<br>KO-350: 0.4	KO-Veh: 0.4<br>KO-350: 0.1	KO-Veh: 0.4<br>KO-350: 0.5	KO-Veh: 0.9<br>KO-350: 0.6	KO-Veh: 1.2<br>KO-350: 0.6	KO-Veh: 0.7<br>KO-350: 0.5
N (animals)	KO-Veh: 5<br>KO-350: 8	KO-Veh: 5<br>KO-350: 8	KO-Veh: 5<br>KO-350: 8	KO-Veh: 5<br>KO-350: 8	KO-Veh: 5<br>KO-350: 8	KO-Veh: 5<br>KO-350: 8	KO-Veh: 5<br>KO-350: 8	KO-Veh: 5<br>KO-350: 8

**Fig. 4e: Total Baseline EEG Power**			
**Quantitative Measurements**	**WT-Veh vs. KO-Veh**	**WT-Veh vs. KO-350**	**KO-Veh vs. KO-350**
2-way ANOVA: *p-value*	0.04	0.99	0.02
Mean (μV^2)	WT-Veh: 0.253<br>KO-Veh: 0.427	WT-Veh: 0.253<br>KO-350: 0.268	KO-Veh: 0.427<br>KO-350: 0.268
Standard Error of Mean	WT-Veh: 0.030<br>KO-Veh: 0.056	WT-Veh: 0.030<br>KO-350: 0.032	KO-Veh: 0.056<br>KO-350: 0.032
N (animals)	WT-Veh: 8<br>KO-Veh: 10	WT-Veh: 8<br>KO-350: 12	KO-Veh:10<br>KO-350:12
**Fig. 4f: Delta EEG Power**			
**Quantitative Measurements**	**WT-Veh vs. KO-Veh**	**WT-Veh vs. KO-350**	**KO-Veh vs. KO-350**
2-way ANOVA: *p-value*	0.04	0.96	0.01
Mean (μV^2)	WT-Veh: 0.043<br>KO-Veh: 0.106	WT-Veh: 0.043<br>KO-350: 0.046	KO-Veh: 0.106<br>KO-350: 0.046
Standard Error of Mean	WT-Veh: 0.010<br>KO-Veh: 0.020	WT-Veh: 0.010<br>KO-350: 0.008	KO-Veh: 0.020<br>KO-350: 0.008
N (animals)	WT-Veh: 8<br>KO-Veh: 10	WT-Veh: 8<br>KO-350: 12	KO-Veh:10<br>KO-350:12
**Fig. 4f: Theta EEG Power**			
**Quantitative Measurements**	**WT-Veh vs. KO-Veh**	**WT-Veh vs. KO-350**	**KO-Veh vs. KO-350**
2-way ANOVA: *p-value*	0.04	0.43	0.67
Mean (μV^2)	WT-Veh: 0.028<br>KO-Veh: 0.047	WT-Veh: 0.028<br>KO-350: 0.039	KO-Veh: 0.047<br>KO-350: 0.039
Standard Error of Mean	WT-Veh: 0.006<br>KO-Veh: 0.003	WT-Veh: 0.006<br>KO-350: 0.005	KO-Veh: 0.003<br>KO-350: 0.005
N (animals)	WT-Veh: 8<br>KO-Veh: 10	WT-Veh: 8<br>KO-350: 12	KO-Veh:10<br>KO-350:12
**Fig. 4f: Alpha EEG Power**			

Quantitative Measurements	WT-Veh vs. KO-Veh	WT-Veh vs. KO-350	KO-Veh vs. KO-350
2-way ANOVA: *p-value*	0.37	0.99	0.26
Mean (μV^2)	WT-Veh: 0.014<br>KO-Veh: 0.019	WT-Veh: 0.014<br>KO-350: 0.014	KO-Veh: 0.019<br>KO-350: 0.014
Standard Error of Mean	WT-Veh: 0.002<br>KO-Veh: 0.002	WT-Veh: 0.002<br>KO-350: 0.001	KO-Veh: 0.002<br>KO-350:0.001
N (animals)	WT-Veh: 8<br>KO-Veh: 10	WT-Veh: 8<br>KO-350: 12	KO-Veh:10<br>KO-350:12
**Fig. 4f: Beta EEG Power**			
**Quantitative Measurements**	**WT-Veh vs. KO-Veh**	**WT-Veh vs. KO-350**	**KO-Veh vs. KO-350**
2-way ANOVA: *p-value*	0.57	0.37	0.04
Mean (μV^2)	WT-Veh: 0.004<br>KO-Veh: 0.006	WT-Veh: 0.004<br>KO-350: 0.004	KO-Veh: 0.004<br>KO-350: 0.004
Standard Error of Mean	WT-Veh: 0.0007<br>KO-Veh: 0.001	WT-Veh: 0.0007<br>KO-350: 0.0005	KO-Veh: 0.001<br>KO-350: 0.0005
N (animals)	WT-Veh: 8<br>KO-Veh: 10	WT-Veh: 8<br>KO-350: 12	KO-Veh:10<br>KO-350:12
**Fig. 5e: Latency to Seizure**			
**Quantitative Measurements**	**WT-Veh vs. KO-Veh**	**WT-Veh vs. KO-350**	**KO-Veh vs. KO-350**
2-way ANOVA: *p-value*	0.01	0.99	0.01
Mean (minutes)	WT-Veh: 24.75<br>KO-Veh: 15.6	WT-Veh: 24.75<br>KO-350: 24.25	KO-Veh: 15.6<br>KO-350: 24.25
Standard Error of Mean	WT-Veh: 1.37<br>KO-Veh: 1.79	WT-Veh: 1.37<br>KO-350: 1.98	KO-Veh: 1.79<br>KO-350: 1.98
N (animals)	WT-Veh: 8<br>KO-Veh: 10	WT-Veh: 8<br>KO-350: 12	KO-Veh:10<br>KO-350:12
**Fig. 5f: Latency to SE**			
**Quantitative Measurements**	**WT-Veh vs. KO-Veh**	**WT-Veh vs. KO-350**	**KO-Veh vs. KO-350**
2-way ANOVA: *p-value*	0.001	0.02	0.01
Mean (minutes)	WT-Veh: 70.88<br>KO-Veh: 44.82	WT-Veh: 70.88<br>KO-350: 58.64	KO-Veh: 44.82<br>KO-350: 58.64
Standard Error of Mean	WT-Veh: 3.020<br>KO-Veh: 2.773	WT-Veh: 3.020<br>KO-350: 2.531	KO-Veh: 2.773<br>KO-350: 2.531
N (animals)	WT-Veh: 8<br>KO-Veh: 9	WT-Veh: 8<br>KO-350: 11	KO-Veh: 9<br>KO-350:11

Fig. 5g: % Epileptic Activity			
**Quantitative Measurements**	**WT-Veh vs. KO-Veh**	**WT-Veh vs. KO-350**	**KO-Veh vs. KO-350**
2-way ANOVA: *p-value*	0.01	0.99	0.01
Mean (minutes)	WT-Veh: 24.75<br>KO-Veh: 15.6	WT-Veh: 24.75<br>KO-350: 24.25	KO-Veh: 15.6<br>KO-350: 24.25
Standard Error of Mean	WT-Veh: 1.37<br>KO-Veh: 1.79	WT-Veh: 1.37<br>KO-350: 1.98	KO-Veh: 1.79<br>KO-350: 1.98
N (animals)	WT-Veh: 8<br>KO-Veh: 10	WT-Veh: 8<br>KO-350: 12	KO-Veh:10<br>KO-350:12
**Fig. 5h: Epileptic Power**			
**Quantitative Measurements**	**WT-Veh vs. KO-Veh**	**WT-Veh vs. KO-350**	**KO-Veh vs. KO-350**
2-way ANOVA: *p-value*	0.89	0.22	0.41
Mean (μV^2)	WT-Veh: 1.666<br>KO-Veh: 1.552	WT-Veh: 1.666<br>KO-350: 1.246	KO-Veh: 1.552<br>KO-350: 1.246
Standard Error of Mean	WT-Veh: 0.1267<br>KO-Veh: 0.1984	WT-Veh: 0.1267<br>KO-350: 0.2030	KO-Veh: 0.1984<br>KO-350: 0.2030
N (animals)	WT-Veh: 8<br>KO-Veh: 8	WT-Veh: 8<br>KO-350: 11	KO-Veh: 8<br>KO-350:11
**Fig. 5i: Percentage Change in Post DZ Power (μV^2)**			
**Quantitative Measurements**	**WT-Veh vs. KO-Veh**	**WT-Veh vs. KO-350**	**KO-Veh vs. KO-350**
2-way ANOVA: *p-value*	0.59	0.10	0.01
Mean (%)	WT-Veh: -47.24<br>KO-Veh: -30.11	WT-Veh: -47.42<br>KO-350: -93.89	KO-Veh: -30.11<br>KO-350: -93.89
Standard Error of Mean	WT-Veh: 10.19<br>KO-Veh: 11.31	WT-Veh: 10.19<br>KO-350: 7.272	KO-Veh: 11.31<br>KO-350: 7.272
N (animals)	WT-Veh: 8<br>KO-Veh: 8	WT-Veh: 8<br>KO-350: 11	KO-Veh: 8<br>KO-350:11
**Fig. 6c: Sociability, Time in Stranger zone**			
**Quantitative Measurements**	**WT-Veh vs. KO-Veh**	**WT-Veh vs. KO-350**	**KO-Veh vs. KO-350**
2-way ANOVA: *p-value*	0.003	0.54	0.01
Mean (seconds)	WT-Veh: 132.2<br>KO-Veh: 74.30	WT-Veh: 132.2<br>KO-350: 109.7	KO-Veh: 74.30<br>KO-350: 109.7
Standard Error of Mean	WT-Veh: 13.51<br>KO-Veh: 8.243	WT-Veh: 13.51<br>KO-350: 10.39	KO-Veh: 8.243<br>KO-350: 10.39
N (animals)	WT-Veh: 11	WT-Veh: 11	KO-Veh: 18

	KO-Veh: 18	KO-350: 16	KO-350:16
**Fig. 6d: Preference for Sociability, Stranger Zone vs. Dummy Zone**			
**Quantitative Measurements**	**WT-Veh**	**KO-Veh**	**KO-350**
2-way ANOVA: *p-value*	0.01	0.25	0.01
Mean (seconds)	Stranger Zone: 132.2<br>Dummy Zone: 84.61	Stranger Zone: 74.3<br>Dummy Zone: 60.8	Stranger Zone: 106.8<br>Dummy Zone: 69.2
Standard Error of Mean	Stranger Zone: 13.5<br>Dummy Zone: 7.77	Stranger Zone: 8.24<br>Dummy Zone: 6.05	Stranger Zone: 0.173<br>Dummy Zone: 6.01
N (animals)	WT-Veh: 11	KO-Veh: 18	KO-350: 16
**Fig. 7c: Latency to Escape Hole (s), WT-Veh**			
**Quantitative Measurements**	**Day 1 vs. Day 2**	**Day 1 vs. Day 3**	**Day 1 vs. Day 4**
Repeated Measures ANOVA: *p-value*	<0.0001	<0.0001	<0.0001
Mean (seconds)	Day 1: 147.6<br>Day 2: 79.06	Day 1: 147.6<br>Day 3: 34.77	Day 1: 147.6<br>Day 4: 34.77
Standard Error of Mean	Day 1: 9.095<br>Day 2: 8.926	Day 1: 9.095<br>Day 3: 4.465	Day 1: 9.095<br>Day 4: 4.773
N (animals)	WT-Veh: 11	WT-Veh: 11	WT-Veh: 11
**Fig. 7d: Latency to Escape Hole (s), KO-Veh**			
**Quantitative Measurements**	**Day 1 vs. Day 2**	**Day 1 vs. Day 3**	**Day 1 vs. Day 4**
Repeated Measures ANOVA: *p-value*	0.95	0.42	0.02
Mean (seconds)	Day 1: 136.3<br>Day 2: 140.4	Day 1: 136.3<br>Day 3: 124.9	Day 1: 136.3<br>Day 4: 105.1
Standard Error of Mean	Day 1: 9.720<br>Day 2: 7.301	Day 1: 9.720<br>Day 3: 10.65	Day 1: 9.720<br>Day 4: 5.753
N (animals)	KO-Veh: 18	KO-Veh: 18	KO-Veh: 18
**Fig. 7e: Latency to Escape Hole (s), KO-350**			
**Quantitative Measurements**	**Day 1 vs. Day 2**	**Day 1 vs. Day 3**	**Day 1 vs. Day 4**
Repeated Measures ANOVA: *p-value*	0.44	<0.0001	<0.0001
Mean (seconds)	Day 1: 132.8<br>Day 2: 115.2	Day 1: 132.8<br>Day 3: 60.40	Day 1: 132.8<br>Day 4: 66.66
Standard Error of Mean	Day 1: 10.12<br>Day 2: 7.737	Day 1: 10.12<br>Day 3: 5.603	Day 1: 10.12<br>Day 4: 7.114
N (animals)	KO-350: 16	KO-350: 16	KO-350: 16
**Fig. 7f: Short-term memory: Time spent in Goal Zone**			
**Quantitative**	**WT-Veh vs. KO-Veh**	**WT-Veh vs. KO-350**	**KO-Veh vs. KO-350**

**Measurements**			
2-way ANOVA: p-value	0.003	0.46	0.02
Mean (seconds)	WT-Veh: 17.16<br>KO-Veh: 6.87	WT-Veh: 17.16<br>KO-350: 14.45	KO-Veh: 6.87<br>KO-350: 14.45
Standard Error of Mean	WT-Veh: 2.03<br>KO-Veh: 1.29	WT-Veh: 2.03<br>KO-350: 2.09	KO-Veh: 1.29<br>KO-350: 2.09
N (animals)	WT-Veh: 11<br>KO-Veh: 18	WT-Veh: 11<br>KO-350: 16	KO-Veh: 18<br>KO-350:16

**Fig. 7f: Short-term Memory, Time spent in Arena**				
**Comparisons**	**Genotypes**	Repeated Measures ANOVA: *p-value*	Mean (seconds)	Standard Error of Mean (seconds)
0 vs. 45	WT-Veh=11	0.0001	0= 17.16; 45=1.75	0= 2.03; 45= 0.83
0 vs. 90		0.0005	0= 17.16; 45=1.89	0= 2.03; 90=1.12
0 vs. 135		0.0003	0= 17.16; 135=1.70	0= 2.03; 135=0.91
0 vs. 180		0.0001	0= 17.16; 180=1.41	0= 2.03; 180= 0.74
0 vs. 225		0.0004	0= 17.16; 225= 1.62	0= 2.03; 225= 0.68
0 vs. 270		0.0003	0= 17.16; 270=1.94	0= 2.03; 270= 0.61
0 vs. 315		0.0003	0= 17.16; 315=1.65	0= 2.03; 315= 0.71
0 vs. 45	KO-Veh=18	0.9963	0=6.87; 45=7.46	0=1.29; 45=1.31
0 vs. 90		0.1079	0=6.87; 90=3.16	0=1.29; 90=0.88
0 vs. 135		0.0009	0=6.87; 135=0.79	0=1.29; 135=0.27
0 vs. 180		0.0010	0=6.87; 180=0.83	0=1.29; 180=0.26
0 vs. 225		0.0033	0=6.87; 225=1.67	0=1.29; 225=0.67
0 vs. 270		0.0101	0=6.87; 270=2.07	0=1.29; 270=0.58
0 vs. 315		0.5738	0=6.87; 315= 4.59	0=1.29; 315= 1.04
0 vs. 45	KO-350=16	0.7123	0=14.45; 45=11.39	0=2.09; 45=3.00
0 vs. 90		0.0133	0=14.45; 90=6.55	0=2.09; 90=1.69
0 vs. 135		<0.0001	0=14.45; 135=1.97	0=2.09; 135=0.65
0 vs. 180		<0.0001	0=14.45; 180=1.09	0=2.09; 180=0.38
0 vs. 225		<0.0001	0=14.45; 225=1.41	0=2.09; 225=0.37
0 vs. 270		0.0023	0=14.45; 270=5.35	0=2.09; 270=0.98
0 vs. 315		0.0012	0=14.45; 315=6.37	0=2.09; 315=1.13

**Fig. 7g: Long-term memory: Time spent in Goal Zone**			
**Quantitative Measurements**	**WT-Veh vs. KO-Veh**	**WT-Veh vs. KO-350**	**KO-Veh vs. KO-350**
2-way ANOVA: *p-value*	0.01	0.22	0.29
Mean (seconds)	WT-Veh: 11.55<br>KO-Veh: 5.55	WT-Veh: 11.55<br>KO-350: 8.31	KO-Veh: 5.55<br>KO-350: 8.31
Standard Error of Mean	WT-Veh: 1.69<br>KO-Veh: 1.02	WT-Veh: 1.69<br>KO-350: 1.59	KO-Veh: 1.02<br>KO-350: 1.59
N (animals)	WT-Veh: 11<br>KO-Veh: 18	WT-Veh: 11<br>KO-350: 16	KO-Veh: 18<br>KO-350:16

**Fig. 7g: Long-term Memory, Time spent in Arena**				
**Comparisons**	**Genotypes**	Repeated Measures ANOVA: *p-value*	Mean (seconds)	Standard Error of Mean (seconds)
0 vs. 45	WT-Veh=11	0.01	0=10.81; 45=2.18	0=1.91; 45=0.71
0 vs. 90		0.01	0=10.81; 90=2.02	0=1.91; 90=1.02
0 vs. 135		0.01	0=10.81; 135=1.78	0=1.91; 135=0.90
0 vs. 180		0.006	0=10.81; 180=1.22	0=1.91; 180=0.48
0 vs. 225		0.02	0=10.81; 225= 2.64	0=1.91; 225=0.68
0 vs. 270		0.006	0=10.81; 270=1.41	0=1.91; 270=0.48
0 vs. 315		0.03	0=10.81; 315=2.92	0=1.91; 315=0.96
0 vs. 45	KO-Veh=18	0.83	0=6.44; 45=9.81	0=1.27; 45=3.08
0 vs. 90		0.08	0=6.44; 90=3.28	0=1.27; 90=1.18
0 vs. 135		0.03	0=6.44; 135= 1.57	0=1.27; 135=0.51
0 vs. 180		0.01	0=6.44; 180=1.44	0=1.27; 180=0.60
0 vs. 225		0.03	0=6.44; 225=2.23	0=1.27; 225=0.59
0 vs. 270		0.01	0=6.44; 270=2.51	0=1.27; 270=0.61
0 vs. 315		0.35	0=6.44; 315= 4.04	0=1.27; 315=0.96
0 vs. 45	KO-350=16	>0.9	0=8.13; 45=8.65	0=1.59; 45=1.583
0 vs. 90		0.03	0=8.13; 90=2.31	0=1.59; 90=0.56
0 vs. 135		0.01	0=8.13; 135= 0.55	0=1.59; 135=0.24
0 vs. 180		0.01	0=8.13; 180=0.58	0=1.59; 180=0.15
0 vs. 225		0.02	0=8.13; 225=1.03	0=1.59; 225= 0.35
0 vs. 270		0.04	0=8.13; 270=3.95	0=1.59; 270=1.25
0 vs. 315		0.04	0=8.13; 315=4.67	0=1.59; 315=0.74

Supplementary Table 2

Supplementary Fig. 1b			
**Quantitative Measurements**	**WT vs. KO**	**WT vs. KO-HF**	**KO vs. KO-HF**
One-Way ANOVA: Tukey's multiple comparison: *p-value*	0.5837	0.02	0.004
Mean (mV)	WT: -57.28<br>KO: -61.15	WT: -57.28<br>KO-HF: -44.15	KO: -61.15<br>KO-HF: -44.15
Standard Error of Mean	WT: 2.535<br>KO: 2.892	WT: 2.535<br>KO-HF: 3.586	KO: 2.892<br>KO-HF: 3.586
N (animals); n (Cells)	N=WT:5, KO:4<br>n= WT:7, KO:7	N=WT:5, KO-HF:4<br>n= WT:7, KO-HF:4	N= KO:4, KO-HF:4<br>n= KO:7, KO-HF:4
**Supplementary Fig. 1c**			
**Quantitative Measurements**	**WT vs. KO**	**WT vs. KO-HF**	**KO vs. KO-HF**
One-Way ANOVA: Tukey's multiple comparison: *p-value*	0.4427	0.0360	0.0040
Mean (mV)	WT: 11.07<br>KO: 13.95	WT: 11.07<br>KO-HF: 4.11	KO: 13.95<br>KO-HF: 4.11
Standard Error of Mean	WT: 1.085<br>KO: 2.001	WT: 1.085<br>KO-HF: 2.547	KO: 2.001<br>KO-HF: 2.547
N (animals); n (Cells)	N=WT:6, KO: 5<br>n= WT:11, KO:9	N=WT:6, KO: 5<br>n= WT:11, KO-HF:6	N= KO:5, KO-HF:5<br>n= KO:9, KO-HF:6

Supplementary Fig. 2: Day 1			
**Quantitative Measurements**	**WT-Veh vs. KO-Veh**	**WT-Veh vs. KO-350**	**KO-Veh vs. KO-350**
2-way ANOVA: *p-value*	0.96	0.97	0.83
Mean (seconds)	WT-Veh: 147.6<br>KO-Veh: 136.3	WT-Veh: 146.7<br>KO-350: 132.8	KO-Veh: 136.3<br>KO-350: 132.8
Standard Error of Mean	WT-Veh: 9.09<br>KO-Veh: 9.72	WT-Veh: 9.09<br>KO-350: 10.1	KO-Veh: 9.72<br>KO-350: 10.1
N (animals)	WT-Veh: 16<br>KO-Veh: 18	WT-Veh: 11<br>KO-350: 16	KO-Veh: 18<br>KO-350: 16
**Supplementary Fig. 2: Day 2**			
**Quantitative Measurements**	**WT-Veh vs. KO-Veh**	**WT-Veh vs. KO-350**	**KO-Veh vs. KO-350**
2-way ANOVA: *p-value*	0.0009	0.02	0.31
Mean (seconds)	WT-Veh: 79.04<br>KO-Veh: 136.0	WT-Veh: 79.04<br>KO-350: 115.2	KO-Veh: 136.0<br>KO-350: 115.2
Standard Error of Mean	WT-Veh: 8.92	WT-Veh: 8.92	KO-Veh: 7.23

	KO-Veh: 7.23	KO-350: 7.73	KO-350: 7.73
N (animals)	WT-Veh: 11<br>KO-Veh: 18	WT-Veh: 11<br>KO-350: 16	KO-Veh: 18<br>KO-350: 16
**Supplementary Fig. 2: Day 3**			
**Quantitative Measurements**	**WT-Veh vs. KO-Veh**	**WT-Veh vs. KO-350**	**KO-Veh vs. KO-350**
2-way ANOVA: *p-value*	<0.0001	0.02	<0.0001
Mean (seconds)	WT-Veh: 34.77<br>KO-Veh: 124.9	WT-Veh: 34.77<br>KO-350: 60.40	KO-Veh: 124.9<br>KO-350: 60.40
Standard Error of Mean	WT-Veh: 4.46<br>KO-Veh: 10.65	WT-Veh: 4.46<br>KO-350: 5.60	KO-Veh: 10.65<br>KO-350: 5.60
N (animals)	WT-Veh: 11<br>KO-Veh: 18	WT-Veh: 11<br>KO-350: 16	KO-Veh: 18<br>KO-350: 16
**Supplementary Fig. 2: Day 4**			
**Quantitative Measurements**	**WT-Veh vs. KO-Veh**	**WT-Veh vs. KO-350**	**KO-Veh vs. KO-350**
2-way ANOVA: *p-value*	<0.0001	<0.0001	0.0001
Mean (seconds)	WT-Veh: 22.38<br>KO-Veh: 105.1	WT-Veh: 22.38<br>KO-350: 66.66	KO-Veh: 105.1<br>KO-350: 66.66
Standard Error of Mean	WT-Veh: 4.77<br>KO-Veh: 5.75	WT-Veh: 4.77<br>KO-350: 7.11	KO-Veh: 5.75<br>KO-350: 7.11
N (animals)	WT-Veh: 11<br>KO-Veh: 18	WT-Veh: 11<br>KO-350: 16	KO-Veh: 18<br>KO-350: 16
